# Supplementary material for: Altered microRNA Transcriptome in Cultured Human Liver Cells upon Infection with Ebola Virus
Source: Int J Mol Sci. 2021 Apr 6;22(7):3792. doi: 10.3390/ijms22073792 (PMC8038836; doi:10.3390/ijms22073792)
Supplement: Supplementary file 1 [file ijms-22-03792-s001.zip › Supplementary_File/C_ GO_Analysis_Results/16-30nt_go_Mayinga-24h-Huh7_vs_Control-24h-Huh7_up.mature_mirna_targets/MF_result(Human).html]

| GO.ID | Term | Ontology | Count | Pop.Hits | List.Total | Pop.Total | Fold.Enrichment | Pvalue | FDR | Enrichment.Score | Gene.Ratio | GENES |
| --- | --- | --- | --- | --- | --- | --- | --- | --- | --- | --- | --- | --- |
| GO:0000981 | RNA polymerase II transcription factor activity, sequence-specific DNA binding | Molecular function | 121 | 1108 | 937 | 17548 | 2.04518992560172 | 1.88940577311625e-14 | 2.44866988195866e-11 | 13.7236747619825 | 0.129135538954109 | ETS1//JUN//MEF2D//TEAD1//TFAP2A//FOSL1//CLOCK//CEBPB//KLF6//EGR2//ELK3//ERG//ESR1//FLI1//NR5A2//GRHL1//IRF1//AR//MEOX2//MYB//MYBL1//NEUROD1//NFIA//NFIB//NHLH2//NR4A2//OTX2//POU3F2//POU4F1//BCL11B//SOX4//SOX11//STAT3//TCF21//TEF//TFAP2C//KLF10//NR4A3//HMGA2//KLF7//FUBP3//KLF4//HAND2//TSHZ1//CC2D1B//EN1//MYT1L//ZFPM2//ZBTB7A//CC2D1A//TSHZ3//RBL2//REST//TGIF1//ZNF217//BTG2//BHLHE41//BHLHE40//HAND1//PBX3//ARX//E2F1//PURG//JARID2//ZBTB4//ZEB1//ARID5B//CREBRF//E2F3//FOXJ3//FOXF1//FOXF2//FOXC1//GATA6//MAF//PAX9//RAD21//SOX17//SHOX//RARB//PPARG//THRA//KLF2//IRF9//ZBTB18//ZNF268//KLF12//NKX2-3//DR1//E2F5//EMX2//EZH1//CIC//LHX6//HBP1//HIVEP2//MNX1//HOXA3//HOXD1//FOXN2//BOLA3//NR3C2//LHX8//NEUROG1//NFE2//NPAS2//PLEK//TRERF1//ZNFX1//MIER1//DMRTA1//NKX2-4//RFX7//STAT4//ZNF3//ZNF131//TCF7L1//LCOR//RUNX3//CBFB//ST18// |
| GO:0001067 | regulatory region nucleic acid binding | Molecular function | 102 | 878 | 937 | 17548 | 2.17567334317103 | 5.71870865171258e-14 | 3.70572320630975e-11 | 13.2427020286809 | 0.108858057630736 | CRY2//BAHD1//NR5A2//ZNF568//MEF2D//NFE2//PPARG//RARB//SOX4//SOX11//TFAP2A//THRA//PER2//CEBPB//ZMYND11//CREBRF//E2F3//FOXF1//FOXF2//FOXC1//GATA6//PURG//JARID2//NHLH2//NME1//NR4A2//PAX9//KLF3//ZBTB4//TEF//TFAP2C//NR4A3//ARID5B//RUNX3//KAT2B//HAND2//KLF6//EGR2//CC2D1B//ELK3//EN1//ERG//ESR1//ETS1//FLI1//GRHL1//H2AFZ//IRF1//AR//JUN//MEOX2//MYB//MYBL1//NFIA//NFIB//OTX2//ZBTB7A//POU3F2//CC2D1A//RFX7//BCL11B//SMARCD2//STAT3//TGIF1//KLF10//ZNF217//BHLHE41//FOSL1//KLF7//FUBP3//HAND1//CLOCK//USP3//GADD45A//ARX//PBX3//POU4F1//TEAD1//KLF4//RBL2//REST//IRF9//E2F1//NPAS2//HMGA2//ST18//MBNL1//SLC2A4RG//HIVEP2//ARID4B//ZNF532//TRERF1//ARID4A//SOX17//TNF//TBL1XR1//TCF7L1//NEUROD1//NEUROG1//ZEB1//TCF21//BHLHE40// |
| GO:0044212 | transcription regulatory region DNA binding | Molecular function | 101 | 877 | 937 | 17548 | 2.15679970404588 | 1.33421748427038e-13 | 5.76381953204804e-11 | 12.8747733724285 | 0.107790821771612 | CRY2//BAHD1//NR5A2//ZNF568//MEF2D//NFE2//PPARG//RARB//SOX4//SOX11//TFAP2A//THRA//PER2//CEBPB//ZMYND11//CREBRF//E2F3//FOXF1//FOXF2//FOXC1//GATA6//PURG//JARID2//NHLH2//NME1//NR4A2//PAX9//KLF3//ZBTB4//TEF//TFAP2C//NR4A3//ARID5B//RUNX3//KAT2B//HAND2//KLF6//EGR2//CC2D1B//ELK3//EN1//ERG//ESR1//ETS1//FLI1//GRHL1//H2AFZ//IRF1//AR//JUN//MEOX2//MYB//MYBL1//NFIA//NFIB//OTX2//ZBTB7A//POU3F2//CC2D1A//RFX7//BCL11B//SMARCD2//STAT3//TGIF1//KLF10//ZNF217//BHLHE41//FOSL1//KLF7//FUBP3//HAND1//CLOCK//USP3//GADD45A//ARX//PBX3//POU4F1//TEAD1//KLF4//RBL2//REST//IRF9//E2F1//NPAS2//HMGA2//ST18//SLC2A4RG//NEUROD1//NEUROG1//ZEB1//TCF21//BHLHE40//HIVEP2//ARID4B//ZNF532//TRERF1//ARID4A//SOX17//TNF//TBL1XR1//TCF7L1// |
| GO:0000976 | transcription regulatory region sequence-specific DNA binding | Molecular function | 87 | 740 | 937 | 17548 | 2.20178834116934 | 2.47989940281804e-12 | 8.03487406513045e-10 | 11.6055659359831 | 0.0928495197438634 | CEBPB//ZMYND11//CREBRF//E2F3//FOXF1//FOXF2//FOXC1//GATA6//PURG//JARID2//MEF2D//NHLH2//NME1//NR4A2//PAX9//KLF3//PPARG//ZBTB4//RARB//TEF//TFAP2A//TFAP2C//THRA//NR4A3//ARID5B//RUNX3//KAT2B//HAND2//KLF6//EGR2//CC2D1B//ELK3//EN1//ERG//ESR1//ETS1//FLI1//NR5A2//GRHL1//H2AFZ//IRF1//AR//JUN//MEOX2//MYB//MYBL1//NFIA//NFIB//OTX2//ZBTB7A//POU3F2//CC2D1A//RFX7//BCL11B//SMARCD2//STAT3//TGIF1//KLF10//ZNF217//BHLHE41//FOSL1//KLF7//FUBP3//HAND1//CLOCK//USP3//GADD45A//SOX11//ARX//PBX3//POU4F1//NFE2//TEAD1//KLF4//REST//SOX4//SLC2A4RG//HMGA2//NEUROD1//NEUROG1//ZEB1//TCF21//BHLHE40//CRY2//BAHD1//ZNF568//PER2// |
| GO:0000982 | transcription factor activity, RNA polymerase II proximal promoter sequence-specific DNA binding | Molecular function | 59 | 415 | 937 | 17548 | 2.66251430481799 | 5.19429041799044e-12 | 1.34636007634312e-09 | 11.2844737721875 | 0.0629669156883671 | CEBPB//KLF6//EGR2//ELK3//ERG//ESR1//ETS1//FLI1//NR5A2//GRHL1//IRF1//AR//JUN//MEF2D//MEOX2//MYB//MYBL1//NEUROD1//NFIA//NFIB//NHLH2//NR4A2//OTX2//POU3F2//POU4F1//BCL11B//SOX4//SOX11//STAT3//TCF21//TEF//TFAP2A//TFAP2C//KLF10//NR4A3//FOSL1//HMGA2//KLF7//FUBP3//KLF4//HAND2//TSHZ1//CC2D1B//EN1//MYT1L//ZFPM2//ZBTB7A//CC2D1A//TSHZ3//RBL2//REST//TGIF1//ZNF217//BTG2//BHLHE41//BHLHE40//HAND1//TEAD1//CLOCK// |
| GO:0005515 | protein binding | Molecular function | 711 | 11565 | 937 | 17548 | 1.15136228297115 | 6.73961068167581e-12 | 1.45575590724198e-09 | 11.1713651900646 | 0.75880469583778 | STX6//STX2//NSF//GTF2E1//WWP2//ZFPM2//GATA6//AR//TEAD1//KLF4//ESR1//EGR2//JUN//MEF2D//NEUROD1//NHLH2//RBL2//BHLHE41//BHLHE40//TCERG1//RBBP8//STAT3//BDP1//PER2//FOXC1//NR4A3//S1PR1//GNAQ//CRK//CRKL//GRB2//MAPK1//PIK3R3//ITGAV//DRD1//LPAR1//LPAR3//COL1A2//F3//LDLR//TNF//ZMAT3//CORO2B//IQGAP2//WASF3//KLHL2//FGD4//CNN3//EPS8//PALLD//FLII//KLHL3//TMOD3//MSN//NF2//PFN2//SNTG1//PXK//ENAH//TWF1//TMOD1//CALD1//WASL//LIMA1//TOB1//GAB1//ARHGAP1//ARHGAP6//NCK1//MARCKS//PLEK//UGT1A10//UGT1A7//SRSF2//YWHAG//ADAM9//SQSTM1//RAPGEF4//TAGAP//FGD5//RAB3GAP1//DOCK9//ARHGEF4//RALGPS2//ARHGEF28//SOS1//SOS2//DOCK5//LAMTOR3//FNIP1//PSD//RANBP9//ADRB1//EREG//RASGEF1A//NRG1//IL2//JAK1//MET//ADAM10//EFNA1//CADM2//GJA1//IDH1//ITGB8//LAMA3//LRP6//NTF3//ACOX1//PKD2//CCL2//BMP3//CAPRIN2//TGFB2//WNT7A//BAG6//FLRT1//NPTN//BAMBI//DLL1//GDF11//EDN1//IFNA1//INHBB//OSM//ITGA5//SNX2//SOCS5//IGF1//RASL11B//PTEN//EFNA5//CD2AP//SPRED1//NPNT//ITGA6//KDR//PTHLH//TTR//SNAP25//ADCY3//MKNK2//MYO1B//PPP3R1//TRPV6//STRN//WFS1//CEP97//CDC42EP3//LBR//CCL8//CXCL14//CTNND2//AMER2//NR4A2//SOX17//SKP1//TBL1XR1//TCF7L1//BCL2L11//CETN1//DCX//DPYSL2//MAPRE3//FMR1//KIF5A//NEFM//NEFH//SYBU//MDM1//CLIP1//KIF13A//HAUS8//KIF23//TRIM3//NPAT//PIAS4//PPARG//PPP2CA//PRKAA1//MID1IP1//ATXN1//OGN//VEGFB//DYNLL2//CDH20//MYLIP//PTPN4//DR1//E2F1//E2F5//ETS1//FHL2//FOXF2//CNOT7//MDFIC//JARID2//NFIA//TRERF1//RARB//REST//RNF4//ZEB1//THRA//UBE2I//HMGA2//KAT2B//HAND1//HAND2//AKAP11//CALM2//OTX2//RGL1//DUSP10//IPO5//STMN2//VBP1//SPHK2//ACTC1//DENND5B//DENND1B//TRAPPC8//SBF2//KHDRBS3//EPS15//ERRFI1//RUFY2//PLSCR4//CCDC6//ADAM12//QKI//TBC1D20//TBC1D12//EVI5//TBC1D17//USP6NL//FZD10//APCDD1//FRZB//FZD6//EXOC5//DUSP1//BMPR2//LRP8//CDK19//MDM4//NME1//EGLN1//UGT1A1//BTG1//HDHD2//CEBPB//GADD45A//CDKN1A//MAP3K2//OSR1//CCNY//RHOC//RND3//MAP3K5//PPP1R12A//PPP1R12C//ZBTB4//RHEB//CCND2//CCNE2//CAMK2N2//BAG5//CRY2//MTMR9//DDX6//ARFIP1//PRKAR2B//AIDA//ZFYVE9//SYT6//TMED9//NOG//G3BP2//GRIA1//PLEKHA1//SEMA4B//CDK6//FBXW7//SACS//DNAJB9//DNAJB12//PPP4R2//RAB38//CDC37L1//HSPA13//ARAP2//APPL1//AGFG1//AGFG2//PEX5L//GIT2//EPHA2//EPHA4//EPHA5//EPHA7//MAP2K4//TGFBR2//ZFAND6//RNF180//ANKIB1//LONRF3//LONRF1//SPOPL//ATXN3//JKAMP//UBE2W//NPLOC4//PRKACB//MOAP1//AKTIP//UBE2Z//TSG101//ZFAND4//GNB5//P2RY1//ARRDC3//SOBP//TSC1//PRDM16//VLDLR//ACBD3//SNX5//ZBTB7A//ZMYND11//GLYR1//TACC2//SOSTDC1//DKK3//DLC1//ATAD2//DEK//ANP32E//USP3//HMG20A//FCHO2//CLCN5//NAPEPLD//ARL6IP1//GJB2//RASSF3//RGMB//SCHIP1//HPRT1//HSBP1//CLDN11//ST8SIA3//RAB9B//IER5//PON2//MIEF1//POU3F2//ATG16L1//ZBTB26//PTPRG//HCN2//BNIP3//STAT4//UGP2//ZNF3//DCTPP1//ADIPOR2//DPY30//TRIP10//SLIT2//SLK//CALCOCO2//ACOT7//GRHL1//RBM44//FIBIN//NEUROG1//CHMP3//UGT1A8//UGT1A6//UGT1A9//UGT1A4//UGT1A3//DSCAML1//DMRTA1//TFAP2A//UXS1//FXR1//GNPTG//ACVR1//CD69//TOB2//DNMT3B//MIER1//TCF21//BRMS1L//MZT1//UBXN2B//PLAA//NTN4//PIK3CB//PIKFYVE//FYCO1//CCT6A//TOPORS//AHNAK//MB21D2//TES//GOLGA3//PFKP//CC2D1A//PAK6//RPL15//DYNC1LI2//RAET1E//H2AFZ//RRAGD//SOX4//E2F3//SLC24A2//NPAS2//TFAP2C//CLOCK//GLRX//NFE2//NSMF//TGFB1I1//NDFIP2//PMEPA1//CFL2//PALM2//CRYBG3//DUSP2//GOLGA4//POU4F1//PTGES3//DNAJB4//DNAJB5//URI1//MASTL//EDEM1//GSG1//TGIF1//LDLRAD4//BRD3//RNF2//HOXA3//DSC2//TREM1//RGMA//ARRDC4//CHRM2//CDK8//RTN3//KLF2//IRF9//PGRMC2//ZBTB18//SEC23A//POLR3G//DCTN6//RAI2//COPS8//FASTK//BTG3//PTPN21//RASSF8//EGLN3//KLF12//SCRG1//FAM129A//NEDD1//SLAIN1//FRMD6//NAA30//CMKLR1//NUP35//LYPD6//KLF6//VPS37A//SESN3//PDIK1L//SH3D19//CREBRF//FAM91A1//C12ORF50//DCP2//LCA5//SPTSSA//DUSP5//ZFC3H1//ELAVL2//CC2D1B//ELK3//RAB12//EMX2//SMIM14//ERG//FAM13C//ZBTB9//HS3ST5//PPM1E//CPEB3//NTNG1//DZIP1//BAHD1//WDR47//KDM2A//WDFY3//KLHDC10//RAB21//MYCBP2//FLI1//LRCH1//CIC//UBXN4//FBXO28//OTUD3//MORC3//CNOT6L//NR5A2//LCLAT1//FBXO33//CNEP1R1//PAN3//CLIC4//FBXL5//LHX6//LATS2//RGS17//RANBP6//HBP1//SERP1//SESN1//GLRA1//GLRB//GNS//GPM6A//ANKRD52//PPP1R37//FAM126B//C16ORF72//CD274//GSTA2//ST8SIA5//EPN1//SERTAD3//LRP12//NRBF2//HMGB3//FOXN2//NANOS1//NUP43//IRF1//KCNA4//NHLRC2//MIA3//KCNJ3//KCNJ10//CHCHD10//FAM19A1//MAF//MBNL1//MEOX2//MLLT6//NR3C2//MMP2//LHX8//MPP1//ASPH//MTRR//MYB//ACO1//MINK1//DCAF8//PAX9//DERL2//MEMO1//RLIM//PAIP2//KLF3//DNAJC27//CDK17//ERGIC2//PRR16//TAOK3//YTHDF2//RASD1//ZFR//RAB23//ARID4B//PIGA//PMAIP1//TMX3//ESRP1//DUS2//PLEKHB2//ARMC1//TMEM100//PPP6C//CDCA7L//NAGK//INTS8//MAP7D1//LMBR1L//DCAF6//LMO3//FSTL5//FEM1C//FAM20C//TMEM159//ANKRD50//PTGER4//RAB22A//CNOT6//ZNF512B//HECW2//SLAIN2//RANBP10//TSHZ3//STIM2//USP28//RBM25//RAD21//RAP1B//RBBP7//REV3L//RGS3//RRM2//RTN1//SMOC1//HERPUD2//SHOX//USP46//NABP1//BCL11B//SLC2A4//SMARCD2//ELOVL4//BTF3//TEF//KLF10//TMF1//TRPC3//ZNF217//PTP4A1//BTG2//ZNF655//ZFYVE21//APOO//CENPO//RNF128//FAM118B//PLEKHF2//CLIP4//SNIP1//UBA5//CYBRD1//ELOVL7//C16ORF70//KLHL15//CPEB4//FOSL1//C1ORF21//VANGL1//CAMLG//FAM160A2//ARID5B//KIAA1109//PCGF5//RECK//NSMAF//LCOR//KBTBD8//TMEM185A//TMEM25//PPP1R15B//FAM104A//NUDCD1//PPFIA1//CDC14A//RUNX3//CBFB//SLC4A4//CDC23//AP1S2//CCNC//FUBP3//RPS6KA4//C5ORF30//MCFD2//SESTD1//BTF3L4//ANKRD44//WDR20//RPS6KA5//ORMDL3//ITM2B//CEP41//FEZ2//PPM1F//PHF14//ULK2//SECISBP2L//LAPTM4A//KMT2B//EFCAB14//MFAP3L//TECPR2//JOSD1//GFPT2// |
| GO:1990837 | sequence-specific double-stranded DNA binding | Molecular function | 87 | 776 | 937 | 17548 | 2.09964352121819 | 3.18800931465931e-11 | 5.87008252100213e-09 | 10.4964804183615 | 0.0928495197438634 | CRY2//BAHD1//NR5A2//ZNF568//MEF2D//NFE2//PPARG//RARB//SOX4//SOX11//TFAP2A//THRA//PER2//CEBPB//ZMYND11//CREBRF//E2F3//FOXF1//FOXF2//FOXC1//GATA6//PURG//JARID2//NHLH2//NME1//NR4A2//PAX9//KLF3//ZBTB4//TEF//TFAP2C//NR4A3//ARID5B//RUNX3//KAT2B//HAND2//KLF6//EGR2//CC2D1B//ELK3//EN1//ERG//ESR1//ETS1//FLI1//GRHL1//H2AFZ//IRF1//AR//JUN//MEOX2//MYB//MYBL1//NFIA//NFIB//OTX2//ZBTB7A//POU3F2//CC2D1A//RFX7//BCL11B//SMARCD2//STAT3//TGIF1//KLF10//ZNF217//BHLHE41//FOSL1//KLF7//FUBP3//HAND1//CLOCK//USP3//GADD45A//ARX//PBX3//POU4F1//TEAD1//KLF4//REST//SLC2A4RG//HMGA2//NEUROD1//NEUROG1//ZEB1//TCF21//BHLHE40// |
| GO:0140110 | transcription regulator activity | Molecular function | 168 | 1921 | 937 | 17548 | 1.63783426121556 | 3.73239198883878e-11 | 5.87008252100213e-09 | 10.4280127514074 | 0.179295624332978 | TSHZ1//KLF2//IRF9//ZBTB18//CEBPB//ZNF268//KLF12//KLF6//CREBRF//NKX2-3//ARX//DR1//E2F1//E2F3//E2F5//EGR2//CC2D1B//ELK3//EMX2//EN1//ERG//ESR1//ETS1//EZH1//FOXJ3//FOXF1//FOXF2//FOXC1//MYT1L//FLI1//CIC//ZFPM2//NR5A2//GATA6//LHX6//HBP1//GRHL1//HIVEP2//MNX1//HOXA3//HOXD1//FOXN2//IRF1//AR//JUN//BOLA3//MAF//MEF2D//MEOX2//NR3C2//LHX8//MYB//MYBL1//NEUROD1//NEUROG1//NFIA//NFE2//NFIB//NHLH2//NPAS2//NR4A2//OTX2//PAX9//PBX3//ZBTB7A//PLEK//POU3F2//POU4F1//PPARG//CC2D1A//TRERF1//ZNFX1//TSHZ3//ZBTB4//MIER1//RARB//REST//DMRTA1//SOX17//NKX2-4//SHOX//RFX7//BCL11B//SOX4//SOX11//STAT3//STAT4//ZEB1//TCF21//TEAD1//TEF//TFAP2A//TFAP2C//TGIF1//THRA//KLF10//ZNF3//ZNF131//BHLHE41//NR4A3//FOSL1//TCF7L1//LCOR//BHLHE40//RUNX3//CBFB//KLF4//HAND1//HAND2//CLOCK//ST18//CRY2//HMGA2//PER2//BDP1//GTF2E1//KLF7//FUBP3//RBL2//ZNF217//BTG2//TCERG1//RBBP8//URI1//WWP2//CNOT7//PURG//JARID2//ARID5B//RAD21//HMG20A//ZNF782//ZNF367//ZNF568//LHX1//KLF3//ZNF532//SLC2A4RG//ZNF630//ZNF287//ARID4A//RNF4//PRDM16//ZNF2//ZNF655//BTAF1//KMT2B//ZMYND11//FHL2//NRG1//BTG1//TMF1//KAT2B//TADA1//NPAT//RAP2C//SMARCD2//TGFB1I1//TOB1//TOB2//DNMT3B//HSBP1//RLIM//PIAS4//SRSF2//TSG101//TBL1XR1//DCAF6// |
| GO:0001012 | RNA polymerase II regulatory region DNA binding | Molecular function | 78 | 665 | 937 | 17548 | 2.1966506447549 | 4.07644619514037e-11 | 5.87008252100213e-09 | 10.3897182855386 | 0.0832443970117396 | CEBPB//ZMYND11//CREBRF//E2F3//FOXF1//FOXF2//FOXC1//GATA6//PURG//JARID2//MEF2D//NHLH2//NME1//NR4A2//PAX9//KLF3//PPARG//ZBTB4//RARB//TEF//TFAP2A//TFAP2C//THRA//NR4A3//ARID5B//RUNX3//KAT2B//HAND2//KLF6//EGR2//CC2D1B//ELK3//EN1//ERG//ESR1//ETS1//FLI1//NR5A2//GRHL1//H2AFZ//IRF1//AR//JUN//MEOX2//MYB//MYBL1//NFIA//NFIB//OTX2//ZBTB7A//POU3F2//CC2D1A//RFX7//BCL11B//SMARCD2//STAT3//TGIF1//KLF10//ZNF217//BHLHE41//FOSL1//KLF7//FUBP3//HAND1//CLOCK//USP3//GADD45A//SOX11//ARX//PBX3//POU4F1//HMGA2//NEUROD1//NEUROG1//ZEB1//TCF21//BHLHE40//RBL2// |
| GO:0043565 | sequence-specific DNA binding | Molecular function | 113 | 1139 | 937 | 17548 | 1.8579873562066 | 7.5317498485887e-11 | 9.65794000615055e-09 | 10.1231041126041 | 0.12059765208111 | CRY2//BAHD1//NR5A2//ZNF568//MEF2D//NFE2//PPARG//RARB//SOX4//SOX11//TFAP2A//THRA//PER2//CEBPB//ZMYND11//CREBRF//E2F3//FOXF1//FOXF2//FOXC1//GATA6//PURG//JARID2//NHLH2//NME1//NR4A2//PAX9//KLF3//ZBTB4//TEF//TFAP2C//NR4A3//ARID5B//RUNX3//KAT2B//HAND2//KLF6//EGR2//CC2D1B//ELK3//EN1//ERG//ESR1//ETS1//FLI1//GRHL1//H2AFZ//IRF1//AR//JUN//MEOX2//MYB//MYBL1//NFIA//NFIB//OTX2//ZBTB7A//POU3F2//CC2D1A//RFX7//BCL11B//SMARCD2//STAT3//TGIF1//KLF10//ZNF217//BHLHE41//FOSL1//KLF7//FUBP3//HAND1//CLOCK//USP3//GADD45A//ARX//PBX3//POU4F1//TEAD1//KLF4//REST//SLC2A4RG//HMGA2//NEUROD1//NEUROG1//ZEB1//TCF21//BHLHE40//KLF2//ZBTB18//MTPN//NKX2-3//E2F1//EMX2//FOXJ3//LHX6//GTF2E1//HIVEP2//MNX1//HOXA3//HOXD1//FOXN2//LHX1//MAF//NR3C2//LHX8//ZNF532//DMRTA1//PRDM16//SOX17//NKX2-4//SHOX//SNAPC1//TCF7L1// |
| GO:0000977 | RNA polymerase II regulatory region sequence-specific DNA binding | Molecular function | 77 | 662 | 937 | 17548 | 2.17831544396689 | 8.19732562250433e-11 | 9.65794000615055e-09 | 10.0863278130837 | 0.0821771611526147 | CEBPB//KLF6//EGR2//CC2D1B//ELK3//EN1//ERG//ESR1//ETS1//FLI1//NR5A2//GATA6//GRHL1//H2AFZ//IRF1//AR//JUN//MEF2D//MEOX2//MYB//MYBL1//NFIA//NFIB//NHLH2//NR4A2//OTX2//ZBTB7A//POU3F2//CC2D1A//RFX7//BCL11B//SMARCD2//STAT3//TFAP2A//TGIF1//KLF10//ZNF217//BHLHE41//NR4A3//FOSL1//KLF7//FUBP3//HAND1//CLOCK//USP3//GADD45A//SOX11//ARX//PBX3//POU4F1//HMGA2//NEUROD1//NEUROG1//ZEB1//TCF21//BHLHE40//HAND2//ZMYND11//CREBRF//E2F3//FOXF1//FOXF2//FOXC1//PURG//JARID2//NME1//PAX9//KLF3//PPARG//ZBTB4//RARB//TEF//TFAP2C//THRA//ARID5B//RUNX3//KAT2B// |
| GO:0001228 | transcriptional activator activity, RNA polymerase II transcription regulatory region sequence-specific DNA binding | Molecular function | 55 | 417 | 937 | 17548 | 2.47010076037356 | 4.97825045732441e-10 | 5.08155207451362e-08 | 9.30292325768771 | 0.0586979722518677 | CEBPB//KLF6//EGR2//ELK3//ERG//ESR1//ETS1//FLI1//NR5A2//GRHL1//IRF1//AR//JUN//MEF2D//MEOX2//MYB//MYBL1//NEUROD1//NFIA//NFIB//NHLH2//NR4A2//OTX2//POU3F2//POU4F1//BCL11B//SOX4//SOX11//STAT3//TCF21//TEF//TFAP2A//TFAP2C//KLF10//NR4A3//FOSL1//HMGA2//KLF7//FUBP3//KLF4//HAND2//PBX3//CREBRF//E2F3//FOXJ3//FOXF1//FOXF2//FOXC1//GATA6//MAF//PAX9//RAD21//REST//SOX17//SHOX// |
| GO:0003700 | DNA binding transcription factor activity | Molecular function | 139 | 1551 | 937 | 17548 | 1.67838286587577 | 5.09723587721274e-10 | 5.08155207451362e-08 | 9.29266526874568 | 0.148345784418356 | TSHZ1//KLF2//IRF9//ZBTB18//CEBPB//ZNF268//KLF12//KLF6//CREBRF//NKX2-3//ARX//DR1//E2F1//E2F3//E2F5//EGR2//CC2D1B//ELK3//EMX2//EN1//ERG//ESR1//ETS1//EZH1//FOXJ3//FOXF1//FOXF2//FOXC1//MYT1L//FLI1//CIC//ZFPM2//NR5A2//GATA6//LHX6//HBP1//GRHL1//HIVEP2//MNX1//HOXA3//HOXD1//FOXN2//IRF1//AR//JUN//BOLA3//MAF//MEF2D//MEOX2//NR3C2//LHX8//MYB//MYBL1//NEUROD1//NEUROG1//NFIA//NFE2//NFIB//NHLH2//NPAS2//NR4A2//OTX2//PAX9//PBX3//ZBTB7A//PLEK//POU3F2//POU4F1//PPARG//CC2D1A//TRERF1//ZNFX1//TSHZ3//ZBTB4//MIER1//RARB//REST//DMRTA1//SOX17//NKX2-4//SHOX//RFX7//BCL11B//SOX4//SOX11//STAT3//STAT4//ZEB1//TCF21//TEAD1//TEF//TFAP2A//TFAP2C//TGIF1//THRA//KLF10//ZNF3//ZNF131//BHLHE41//NR4A3//FOSL1//TCF7L1//LCOR//BHLHE40//RUNX3//CBFB//KLF4//HAND1//HAND2//CLOCK//ST18//HMGA2//KLF7//FUBP3//RBL2//ZNF217//BTG2//PURG//JARID2//ARID5B//RAD21//HMG20A//ZNF782//ZNF367//CNOT7//ZNF568//LHX1//KLF3//ZNF532//SLC2A4RG//ZNF630//ZNF287//ARID4A//RNF4//PRDM16//ZNF2//ZNF655//BTAF1//KMT2B// |
| GO:0003690 | double-stranded DNA binding | Molecular function | 88 | 860 | 937 | 17548 | 1.91633863642004 | 2.59212731017814e-09 | 2.39956928142205e-07 | 8.58634367226515 | 0.0939167556029883 | CRY2//BAHD1//NR5A2//ZNF568//MEF2D//NFE2//PPARG//RARB//SOX4//SOX11//TFAP2A//THRA//PER2//CEBPB//ZMYND11//CREBRF//E2F3//FOXF1//FOXF2//FOXC1//GATA6//PURG//JARID2//NHLH2//NME1//NR4A2//PAX9//KLF3//ZBTB4//TEF//TFAP2C//NR4A3//ARID5B//RUNX3//KAT2B//HAND2//KLF6//EGR2//CC2D1B//ELK3//EN1//ERG//ESR1//ETS1//FLI1//GRHL1//H2AFZ//IRF1//AR//JUN//MEOX2//MYB//MYBL1//NFIA//NFIB//OTX2//ZBTB7A//POU3F2//CC2D1A//RFX7//BCL11B//SMARCD2//STAT3//TGIF1//KLF10//ZNF217//BHLHE41//FOSL1//KLF7//FUBP3//HAND1//CLOCK//USP3//GADD45A//ARX//PBX3//POU4F1//TEAD1//KLF4//REST//SLC2A4RG//HMGA2//NEUROD1//NEUROG1//ZEB1//TCF21//BHLHE40//HMGB3// |
| GO:0001077 | transcriptional activator activity, RNA polymerase II proximal promoter sequence-specific DNA binding | Molecular function | 41 | 279 | 937 | 17548 | 2.75212203975932 | 3.59280086005265e-09 | 3.10417994308549e-07 | 8.44456685398295 | 0.0437566702241195 | CEBPB//KLF6//EGR2//ELK3//ERG//ESR1//ETS1//FLI1//NR5A2//GRHL1//IRF1//AR//JUN//MEF2D//MEOX2//MYB//MYBL1//NEUROD1//NFIA//NFIB//NHLH2//NR4A2//OTX2//POU3F2//POU4F1//BCL11B//SOX4//SOX11//STAT3//TCF21//TEF//TFAP2A//TFAP2C//KLF10//NR4A3//FOSL1//HMGA2//KLF7//FUBP3//KLF4//HAND2// |
| GO:0000987 | proximal promoter sequence-specific DNA binding | Molecular function | 55 | 451 | 937 | 17548 | 2.28388473852721 | 8.6659435697928e-09 | 7.01941429153217e-07 | 8.06218414329696 | 0.0586979722518677 | CEBPB//KLF6//EGR2//CC2D1B//ELK3//EN1//ERG//ESR1//ETS1//FLI1//NR5A2//GATA6//GRHL1//H2AFZ//IRF1//AR//JUN//MEF2D//MEOX2//MYB//MYBL1//NFIA//NFIB//NHLH2//NR4A2//OTX2//ZBTB7A//POU3F2//CC2D1A//RFX7//BCL11B//SMARCD2//STAT3//TFAP2A//TGIF1//KLF10//ZNF217//BHLHE41//NR4A3//FOSL1//KLF7//FUBP3//HAND1//CLOCK//USP3//HMGA2//NEUROD1//NEUROG1//ZEB1//TCF21//BHLHE40//HAND2//NFE2//TEAD1//KLF4// |
| GO:0000978 | RNA polymerase II proximal promoter sequence-specific DNA binding | Molecular function | 52 | 436 | 937 | 17548 | 2.23359736813762 | 4.64587355740419e-08 | 3.54179537082108e-06 | 7.3329326141804 | 0.0554962646744931 | ESR1//JUN//HMGA2//NEUROD1//NEUROG1//ZEB1//TCF21//BHLHE41//BHLHE40//HAND2//CLOCK//CEBPB//KLF6//EGR2//CC2D1B//ELK3//EN1//ERG//ETS1//FLI1//NR5A2//GATA6//GRHL1//H2AFZ//IRF1//AR//MEF2D//MEOX2//MYB//MYBL1//NFIA//NFIB//NHLH2//NR4A2//OTX2//ZBTB7A//POU3F2//CC2D1A//RFX7//BCL11B//SMARCD2//STAT3//TFAP2A//TGIF1//KLF10//ZNF217//NR4A3//FOSL1//KLF7//FUBP3//HAND1//USP3// |
| GO:0000989 | transcription factor activity, transcription factor binding | Molecular function | 68 | 643 | 937 | 17548 | 1.98055074681613 | 5.20614711641531e-08 | 3.74842592381902e-06 | 7.28348356366731 | 0.0725720384204909 | KLF4//BDP1//GTF2E1//PPARG//RARB//THRA//TCERG1//TRERF1//ZFPM2//NEUROD1//POU3F2//SOX4//SOX11//RBBP8//URI1//TEAD1//MYB//MYBL1//WWP2//JUN//CLOCK//CNOT7//BHLHE41//BHLHE40//ZMYND11//FHL2//NRG1//BTG1//TMF1//CBFB//KAT2B//ZBTB18//TADA1//FOXC1//NFE2//NPAT//POU4F1//RAP2C//RNF4//PRDM16//SOX17//SMARCD2//ZEB1//TFAP2A//TGFB1I1//ARID5B//KLF7//PER2//HAND1//HAND2//TOB1//TOB2//KLF12//DNMT3B//DR1//ELK3//HSBP1//LHX1//RLIM//PIAS4//SRSF2//TGIF1//TSG101//TBL1XR1//DCAF6//CRY2//NEUROG1//HMGA2// |
| GO:0000988 | transcription factor activity, protein binding | Molecular function | 68 | 648 | 937 | 17548 | 1.96526871944873 | 7.02295208585865e-08 | 4.7903925806699e-06 | 7.15348029455085 | 0.0725720384204909 | CRY2//NEUROG1//HMGA2//PER2//KLF4//BDP1//GTF2E1//PPARG//RARB//THRA//TCERG1//TRERF1//ZFPM2//NEUROD1//POU3F2//SOX4//SOX11//RBBP8//URI1//TEAD1//MYB//MYBL1//WWP2//JUN//CLOCK//CNOT7//BHLHE41//BHLHE40//ZMYND11//FHL2//NRG1//BTG1//TMF1//CBFB//KAT2B//ZBTB18//TADA1//FOXC1//NFE2//NPAT//POU4F1//RAP2C//RNF4//PRDM16//SOX17//SMARCD2//ZEB1//TFAP2A//TGFB1I1//ARID5B//KLF7//HAND1//HAND2//TOB1//TOB2//KLF12//DNMT3B//DR1//ELK3//HSBP1//LHX1//RLIM//PIAS4//SRSF2//TGIF1//TSG101//TBL1XR1//DCAF6// |
| GO:0003677 | DNA binding | Molecular function | 187 | 2520 | 937 | 17548 | 1.38972573732446 | 9.32114878102531e-07 | 6.0401044101044e-05 | 6.03053055990836 | 0.19957310565635 | HMGB3//CRY2//BAHD1//NR5A2//ZNF568//MEF2D//NFE2//PPARG//RARB//SOX4//SOX11//TFAP2A//THRA//PER2//CEBPB//ZMYND11//CREBRF//E2F3//FOXF1//FOXF2//FOXC1//GATA6//PURG//JARID2//NHLH2//NME1//NR4A2//PAX9//KLF3//ZBTB4//TEF//TFAP2C//NR4A3//ARID5B//RUNX3//KAT2B//HAND2//KLF6//EGR2//CC2D1B//ELK3//EN1//ERG//ESR1//ETS1//FLI1//GRHL1//H2AFZ//IRF1//AR//JUN//MEOX2//MYB//MYBL1//NFIA//NFIB//OTX2//ZBTB7A//POU3F2//CC2D1A//RFX7//BCL11B//SMARCD2//STAT3//TGIF1//KLF10//ZNF217//BHLHE41//FOSL1//KLF7//FUBP3//HAND1//CLOCK//USP3//GADD45A//ARX//PBX3//POU4F1//TEAD1//KLF4//RBL2//REST//IRF9//E2F1//NPAS2//HMGA2//ST18//SLC2A4RG//RBBP8//NUP35//NABP1//TRERF1//KLF2//ZBTB18//MTPN//NKX2-3//EMX2//FOXJ3//LHX6//GTF2E1//HIVEP2//MNX1//HOXA3//HOXD1//FOXN2//LHX1//MAF//NR3C2//LHX8//NEUROD1//ZNF532//DMRTA1//PRDM16//SOX17//NKX2-4//SHOX//SNAPC1//TCF7L1//ARID4B//ARID4A//TNF//TBL1XR1//NEUROG1//ZEB1//TCF21//BHLHE40//TSHZ1//TOPORS//HMG20A//ZNF268//KLF12//ZNF597//RBFOX3//ZNF782//ZNF800//GLIS3//DNMT3B//DR1//E2F5//ZNF367//ZBTB9//KDM2A//MYT1L//CIC//ZFPM2//HBP1//ZDHHC1//AGFG1//ZBTB41//IMPDH1//LBR//ZBTB34//SYCP3//RNF141//PIAS4//ZFR//ZFAND6//BNC2//BDP1//MAPK1//PHTF2//ZNF630//ZNF287//ZNF512B//TSHZ3//ZBTB26//MIER1//REV3L//RNF4//ATXN1//SOS1//SOS2//STAT4//TMF1//TSG101//ZNF2//ZNF3//ZNF131//ZNF655//DEK//LCOR//GLYR1//ZIC5//CBFB//BTAF1//ZBTB47//KMT2B// |
| GO:0019899 | enzyme binding | Molecular function | 172 | 2301 | 937 | 17548 | 1.39990918523198 | 1.83038129525211e-06 | 0.000112960674221273 | 5.73745843093903 | 0.183564567769477 | GTF2E1//COL1A2//F3//ITGAV//LDLR//TNF//MARCKS//PLEK//UGT1A10//UGT1A7//SRSF2//YWHAG//ADAM9//SQSTM1//RAPGEF4//TAGAP//FGD4//FGD5//RAB3GAP1//DOCK9//ARHGEF4//RALGPS2//ARHGEF28//SOS1//SOS2//DOCK5//LAMTOR3//FNIP1//PSD//RANBP9//ADRB1//EREG//RASGEF1A//GRB2//NRG1//IL2//JAK1//MET//AKAP11//CALM2//RGL1//DUSP10//IPO5//SPHK2//IQGAP2//CDC42EP3//DENND5B//DENND1B//TRAPPC8//SBF2//TBC1D20//TBC1D12//ARHGAP1//NSF//EVI5//TBC1D17//USP6NL//EXOC5//CEBPB//GADD45A//BTG1//BCL2L11//ADAM10//CDKN1A//MAP3K2//OSR1//SPRED1//DCX//E2F1//ESR1//CCNY//MAPRE3//GATA6//RHOC//RND3//MAP3K5//MSN//PPP1R12A//NEFH//ERRFI1//PPP1R12C//ZBTB4//RHEB//STAT3//NR4A3//KAT2B//CCND2//CCNE2//CAMK2N2//BAG5//CRY2//PPP3R1//MAPK1//PPARG//MTMR9//EPS8//TOB1//NCK1//SOCS5//ARAP2//APPL1//AGFG1//AGFG2//PEX5L//GIT2//EPHA2//EPHA4//EPHA5//EPHA7//KDR//MAP2K4//TGFBR2//RNF180//ANKIB1//LONRF3//LONRF1//EGR2//SPOPL//JUN//ATXN3//JKAMP//PIAS4//UBE2W//FBXW7//NPLOC4//PRKACB//PRKAR2B//MOAP1//AKTIP//UBE2Z//TSG101//WFS1//BAG6//FZD6//ZFAND4//ETS1//ZBTB7A//DNMT3B//MEF2D//MIER1//TCF21//BHLHE41//BRMS1L//KLF4//UBE2I//TOPORS//PAK6//DUSP2//GOLGA4//POU4F1//ATG16L1//RRAGD//BNIP3//AR//PKD2//STRN//MASTL//TCERG1//GSG1//PTGES3//CHMP3//PTEN//CRK//GJA1//CDK19//MDM4//NME1//EGLN1//UGT1A1//PLSCR4//HDHD2//HAND1// |
| GO:0003712 | transcription cofactor activity | Molecular function | 56 | 566 | 937 | 17548 | 1.85293263592173 | 6.28220467193254e-06 | 0.00037007896612839 | 5.2018879185515 | 0.0597652081109925 | TCERG1//TRERF1//ZFPM2//NEUROD1//POU3F2//SOX4//SOX11//RBBP8//URI1//ZBTB18//TADA1//FHL2//FOXC1//JUN//NFE2//NPAT//POU4F1//RAP2C//RNF4//PRDM16//SOX17//SMARCD2//ZEB1//TFAP2A//TGFB1I1//ARID5B//KLF7//CBFB//KAT2B//PER2//HAND1//HAND2//TOB1//TOB2//ZMYND11//KLF12//DNMT3B//DR1//ELK3//HSBP1//LHX1//RLIM//PIAS4//SRSF2//TGIF1//TSG101//BHLHE41//TBL1XR1//BHLHE40//PPARG//DCAF6//RARB//THRA//NRG1//BTG1//TMF1// |
| GO:0001078 | transcriptional repressor activity, RNA polymerase II proximal promoter sequence-specific DNA binding | Molecular function | 21 | 141 | 937 | 17548 | 2.78925497854175 | 1.88401799422421e-05 | 0.00106160318283242 | 4.72491495358403 | 0.0224119530416222 | TSHZ1//CEBPB//CC2D1B//EN1//MYT1L//ZFPM2//ZBTB7A//CC2D1A//TSHZ3//RBL2//REST//TCF21//TFAP2A//TFAP2C//TGIF1//ZNF217//BTG2//BHLHE41//HMGA2//BHLHE40//HAND1// |
| GO:0019901 | protein kinase binding | Molecular function | 61 | 660 | 937 | 17548 | 1.73090779728987 | 2.03362399829086e-05 | 0.00109815695907706 | 4.69172934175869 | 0.0651013874066169 | ITGAV//MARCKS//PLEK//UGT1A10//UGT1A7//SRSF2//YWHAG//ADAM9//SQSTM1//DUSP10//TOB1//NRG1//NCK1//SOCS5//EPHA2//EPHA4//EPHA5//EPHA7//KDR//MET//MAPK1//MAP2K4//TGFBR2//PRKAR2B//APPL1//DUSP2//CRK//GJA1//PTEN//BCL2L11//ADAM10//CDKN1A//MAP3K2//OSR1//SPRED1//DCX//E2F1//ESR1//CCNY//MAPRE3//GATA6//GRB2//RHOC//RND3//MAP3K5//MSN//PPP1R12A//NEFH//NSF//ERRFI1//PPP1R12C//ZBTB4//RHEB//STAT3//NR4A3//CALM2//KAT2B//CCND2//CCNE2//CAMK2N2//BAG5// |
| GO:0005488 | binding | Molecular function | 842 | 15006 | 937 | 17548 | 1.05083658461198 | 3.16702875974273e-05 | 0.00164178770905063 | 4.49934799278822 | 0.898612593383138 | ACOT7//ACBD3//ACBD5//STX6//STX2//NSF//HPRT1//REV3L//PEX5L//IDH1//MAP3K5//NME1//PRKACB//PTEN//RHEB//DCTPP1//RPS6KA4//RPS6KA5//HMGB3//BAG5//CPEB2//CPEB3//CPEB1//CPEB4//CRY2//BAHD1//NR5A2//ZNF568//MEF2D//NFE2//PPARG//RARB//SOX4//SOX11//TFAP2A//THRA//PER2//CEBPB//ZMYND11//CREBRF//E2F3//FOXF1//FOXF2//FOXC1//GATA6//PURG//JARID2//NHLH2//NR4A2//PAX9//KLF3//ZBTB4//TEF//TFAP2C//NR4A3//ARID5B//RUNX3//KAT2B//HAND2//KLF6//EGR2//CC2D1B//ELK3//EN1//ERG//ESR1//ETS1//FLI1//GRHL1//H2AFZ//IRF1//AR//JUN//MEOX2//MYB//MYBL1//NFIA//NFIB//OTX2//ZBTB7A//POU3F2//CC2D1A//RFX7//BCL11B//SMARCD2//STAT3//TGIF1//KLF10//ZNF217//BHLHE41//FOSL1//KLF7//FUBP3//HAND1//CLOCK//USP3//GADD45A//ARX//PBX3//POU4F1//TEAD1//KLF4//GTF2E1//RBL2//REST//IRF9//E2F1//NPAS2//HMGA2//ST18//MBNL1//WWP2//ZFPM2//NEUROD1//BHLHE40//TCERG1//RBBP8//BDP1//SLC2A4RG//GRIA1//LDLR//TGFB2//ITM2B//S1PR1//GNAQ//CRK//CRKL//GRB2//MAPK1//PIK3R3//OSBPL5//FCHO2//SYT6//ITGAV//DRD1//LPAR1//LPAR3//UGT1A8//UGT1A7//UGT1A9//UGT1A1//UGT1A3//COL1A2//F3//TNF//ZMAT3//FMR1//FXR1//OSR1//DZIP1//IMPDH1//REXO1//ZNF804A//TSHZ1//TOPORS//HMG20A//KLF2//ZBTB18//ZNF268//KLF12//ZNF597//RBFOX3//ZNF782//ZNF800//GLIS3//DNMT3B//DR1//E2F5//ZNF367//ZBTB9//KDM2A//MYT1L//CIC//HBP1//ZDHHC1//HIVEP2//AGFG1//ZBTB41//LBR//ZBTB34//NEUROG1//SYCP3//RNF141//PIAS4//ZFR//ZFAND6//BNC2//ZNF532//PHTF2//ZNF630//ZNF287//ZNF512B//TSHZ3//ZBTB26//MIER1//ARID4A//RNF4//ATXN1//SOS1//SOS2//STAT4//TMF1//TSG101//ZNF2//ZNF3//ZNF131//ZNF655//DEK//TCF7L1//LCOR//GLYR1//ZIC5//CBFB//BTAF1//ZBTB47//KMT2B//EZH1//ATAD2//PRKAA1//RAD21//RNF2//GMNC//ZEB1//BRD3//URI1//NUP35//NABP1//KHDRBS3//FASTK//CKAP4//PABPC5//RBM33//DDX6//ZFC3H1//ELAVL2//ZCCHC24//MORC3//PAN3//FAM133B//TES//CNOT7//NANOS1//RBM44//IPO5//ACO1//NOVA1//YTHDF2//RBM47//ZNFX1//CNOT6//RBM25//RBBP7//RPL15//SRSF2//ARHGEF28//FNDC3B//CAPRIN2//BTF3//UBE2I//YWHAG//AHNAK//SNIP1//ZCCHC3//UNK//RTCA//CCT6A//QKI//SECISBP2L//G3BP2//MSN//DUS2//PAIP2//ESRP1//CORO2B//IQGAP2//WASF3//KLHL2//FGD4//CNN3//EPS8//PALLD//FLII//KLHL3//TMOD3//NF2//PFN2//SNTG1//PXK//ENAH//TWF1//TMOD1//CALD1//WASL//LIMA1//TOB1//GAB1//ARHGAP1//ARHGAP6//NCK1//MARCKS//PLEK//UGT1A10//ADAM9//SQSTM1//RAPGEF4//TAGAP//FGD5//RAB3GAP1//DOCK9//ARHGEF4//RALGPS2//DOCK5//LAMTOR3//FNIP1//PSD//RANBP9//ADRB1//EREG//RASGEF1A//NRG1//IL2//JAK1//MET//ADAM10//EFNA1//CADM2//GJA1//ITGB8//LAMA3//LRP6//NTF3//ACOX1//PKD2//CCL2//BMP3//WNT7A//BAG6//FLRT1//NPTN//BAMBI//DLL1//GDF11//EDN1//IFNA1//INHBB//OSM//ITGA5//SNX2//SOCS5//IGF1//RASL11B//EFNA5//CD2AP//SPRED1//NPNT//ITGA6//KDR//PTHLH//TTR//SNAP25//PGRMC2//NR3C2//EGLN3//FBXL5//EGLN1//MSMO1//SCD5//CETN1//DSC2//EPS15//SULF1//SLC24A2//CDH20//MICU3//MAN1A1//ASPH//ASPN//PPP3R1//PRRG1//FSTL5//FAM20C//PLSCR4//SLC24A3//STIM2//SMOC1//SMOC2//SPOCK1//VLDLR//LRP8//PRRG4//CALM2//PCDH11Y//CALN1//MCFD2//SLIT2//CD69//EDEM1//EFCAB14//BCL2L11//CDK6//CDK8//CALCOCO2//CDKN1A//RTN3//DLC1//SEC23A//TRIM3//POLR3G//EXOC5//DCTN6//GNB5//PTGES3//CFL2//RAI2//MAP3K2//TOB2//COPS8//BTG3//STMN2//DNAJB4//PTPN21//FZD10//AKAP11//RASSF8//SCRG1//FAM129A//CLCN5//NEDD1//SLAIN1//FRMD6//NAA30//CMKLR1//TBC1D20//LYPD6//RAET1E//VPS37A//UBXN2B//DYNLL2//SESN3//APCDD1//PDIK1L//CTNND2//PPP4R2//SH3D19//FAM91A1//C12ORF50//DCX//DCP2//LCA5//SPTSSA//DPYSL2//DUSP1//DUSP2//DUSP5//EPHA2//PIKFYVE//RAB12//EMX2//SMIM14//EPHA4//EPHA7//AMER2//CCNY//FAM13C//HS3ST5//FHL2//PPM1E//NTNG1//TRAPPC8//WDR47//MAPRE3//WDFY3//KLHDC10//RAB21//MYCBP2//LRCH1//RGL1//UBXN4//ARL6IP1//FBXO28//OTUD3//RAB38//CNOT6L//LCLAT1//FBXO33//CNEP1R1//DNAJB5//CLIC4//APPL1//LHX6//LATS2//RGS17//RANBP6//SNX5//SERP1//SESN1//GLRA1//GLRB//GNS//GOLGA3//GOLGA4//GPM6A//RASSF3//ANKRD52//PPP1R37//FAM126B//RGMB//MKNK2//C16ORF72//MYLIP//CD274//GSTA2//ST8SIA5//EPN1//SERTAD3//LRP12//MDFIC//SCHIP1//NRBF2//HOXA3//HSBP1//FOXN2//SPOPL//NUP43//KCNA4//NHLRC2//MIA3//KCNJ3//KCNJ10//KIF5A//RHOC//RND3//CHCHD10//FAM19A1//MAF//DNAJB9//MDM4//ATXN3//MLLT6//MMP2//LHX8//MPP1//MZT1//MTRR//PPP1R12A//NEFM//NPAT//OGN//CLDN11//P2RY1//MINK1//DCAF8//DERL2//MEMO1//RLIM//RAB9B//DNAJC27//IER5//CDK17//ERGIC2//PRR16//TAOK3//CHMP3//RASD1//RAB23//ARID4B//PIGA//PIK3CB//PMAIP1//ERRFI1//ANKIB1//MIEF1//TMX3//NDFIP2//TMED9//PPP1R12C//PLEKHB2//ATG16L1//PPP2CA//ARMC1//TMEM100//UBE2W//FBXW7//PPP6C//TRPV6//CDCA7L//NAGK//SYBU//INTS8//CDC37L1//NPLOC4//MAP7D1//LMBR1L//PRKAR2B//DCAF6//LMO3//SPHK2//PAK6//FEM1C//PMEPA1//RGMA//TMEM159//ANKRD50//PTGER4//RAB22A//HECW2//ARRDC3//SLAIN2//RANBP10//USP28//PTPN4//PTPRG//MID1IP1//RRAGD//RAP1B//NTN4//PLEKHA1//RGS3//HCN2//RRM2//CLIP1//RTN1//KIF13A//PRDM16//MOAP1//MAP2K4//HERPUD2//SOX17//AKTIP//SHOX//AIDA//USP46//SKP1//SLC2A4//UBE2Z//BMPR2//MTMR9//BNIP3//HSPA13//ELOVL4//STRN//BTG1//TGFB1I1//TGFBR2//TRPC3//TSC1//UGP2//VBP1//VEGFB//WFS1//PTP4A1//EVI5//BTG2//ZFYVE21//APOO//CENPO//FYCO1//RNF128//CEP97//FAM118B//PLEKHF2//TBL1XR1//TBC1D17//CLIP4//LONRF3//UBA5//CYBRD1//ELOVL7//C16ORF70//CCDC6//KLHL15//ADAM12//C1ORF21//VANGL1//SBF2//CAMLG//FZD6//GSG1//HDHD2//FAM160A2//KIAA1109//BRMS1L//PCGF5//RECK//NSMAF//KBTBD8//TMEM185A//DPY30//TMEM25//PPP1R15B//FAM104A//NUDCD1//PPFIA1//CDC14A//SLC4A4//CDC23//AP1S2//CCNC//CCND2//ACVR1//C5ORF30//CCNE2//SESTD1//BTF3L4//ANKRD44//LONRF1//WDR20//ARRDC4//NOG//TRIP10//HAUS8//ZFYVE9//PLAA//ORMDL3//KIF23//CXCL14//CEP41//FEZ2//PPM1F//PHF14//ULK2//LAPTM4A//SLK//GIT2//MFAP3L//TECPR2//JOSD1//GFPT2//ADCY3//MYO1B//PCOLCE2//CDC42EP3//HIPK3//CLCN6//DGKH//DYNC1LI2//EPHA5//ACSL1//CDK19//DSTYK//CMPK1//PFKP//RIMKLB//STK33//ACTC1//MASTL//DGKE//PKDCC//STK17B//ARL5A//RAB30//GLUD2//NKIRAS1//SAR1B//ARL15//RAP2C//RAB28//HAPLN1//ARFIP1//ARAP2//CCL8//NEFH//MDM1//TRERF1//PDE10A//CHRM2//RNF145//NAPEPLD//ZADH2//MARCH4//MARCH7//FBXO30//USP45//ZFAND4//DUSP10//DENND5B//DENND1B//RUFY2//TBC1D12//USP6NL//FRZB//CAMK2N2//NPEPL1//SEMA4B//CRYBG3//MAN2A1//SACS//DNAJB12//AGFG2//RNF180//JKAMP//SOBP//ST8SIA3//TACC2//SOSTDC1//DKK3//NLN//THOP1//ANP32E//GJB2//PON2//ADIPOR2//FIBIN//UGT1A6//UGT1A4//DSCAML1//DMRTA1//UXS1//GNPTG//TCF21//MTPN//NKX2-3//FOXJ3//MNX1//HOXD1//LHX1//NKX2-4//SNAPC1//CRTAP//MB21D2//PPM1L//RNF38//ESCO2//ZNRF2//ENPP4//STEAP2//YPEL2//RNF186//RSBN1//ENPP5//SLC6A1//DDHD1//SNN//ZC3H12C//LMLN//GLRX//VKORC1L1//NSMF//PALM2//PLEKHA3//LDLRAD4//TREM1// |
| GO:0003713 | transcription coactivator activity | Molecular function | 35 | 317 | 937 | 17548 | 2.06774422699467 | 3.83581071448242e-05 | 0.00191200410998816 | 4.41614283207594 | 0.0373532550693703 | ZFPM2//NEUROD1//POU3F2//SOX4//SOX11//PPARG//TRERF1//DCAF6//RARB//THRA//TSG101//ZBTB18//TCERG1//TADA1//FHL2//FOXC1//JUN//NFE2//NPAT//POU4F1//RAP2C//RNF4//PRDM16//SOX17//SMARCD2//ZEB1//TFAP2A//TGFB1I1//ARID5B//KLF7//CBFB//KAT2B//PER2//HAND1//HAND2// |
| GO:0008134 | transcription factor binding | Molecular function | 57 | 620 | 937 | 17548 | 1.72175439804455 | 4.39735935388347e-05 | 0.00211073248986407 | 4.35680804221936 | 0.0608324439701174 | WWP2//ZFPM2//GATA6//AR//TEAD1//KLF4//ESR1//GTF2E1//EGR2//JUN//MEF2D//NEUROD1//NHLH2//RBL2//BHLHE41//BHLHE40//TCERG1//RBBP8//STAT3//BDP1//PER2//FOXC1//NR4A3//DR1//THRA//PPARG//STRN//DLL1//MDFIC//PRDM16//WFS1//HAND2//TACC2//CEBPB//NR4A2//TOB2//TCF21//HAND1//RARB//FHL2//RNF4//TGFB1I1//E2F1//E2F5//ETS1//FOXF2//CNOT7//JARID2//NFIA//TRERF1//MAPK1//REST//SOX17//ZEB1//UBE2I//HMGA2//KAT2B// |
| GO:0001227 | transcriptional repressor activity, RNA polymerase II transcription regulatory region sequence-specific DNA binding | Molecular function | 28 | 233 | 937 | 17548 | 2.25055766508948 | 5.10664025715124e-05 | 0.00236364491902429 | 4.29186473541586 | 0.0298826040554963 | TSHZ1//CEBPB//CC2D1B//EN1//MYT1L//ZFPM2//ZBTB7A//CC2D1A//TSHZ3//RBL2//REST//TCF21//TFAP2A//TFAP2C//TGIF1//ZNF217//BTG2//BHLHE41//HMGA2//BHLHE40//HAND1//ARX//E2F1//PURG//JARID2//ZBTB4//ZEB1//ARID5B// |
| GO:0015020 | glucuronosyltransferase activity | Molecular function | 9 | 34 | 937 | 17548 | 4.95737334421495 | 5.30805645031762e-05 | 0.00237215212400401 | 4.27506446713181 | 0.0096051227321238 | UGT1A10//UGT1A8//UGT1A7//UGT1A6//UGT1A5//UGT1A9//UGT1A4//UGT1A1//UGT1A3// |
| GO:0003682 | chromatin binding | Molecular function | 51 | 540 | 937 | 17548 | 1.76874184750385 | 5.55632000957343e-05 | 0.00240033024413572 | 4.25521274954535 | 0.0544290288153682 | GRHL1//H2AFZ//STAT3//THRA//CLOCK//RNF4//SMARCD2//HMGA2//FOXC1//RBL2//KLF4//USP3//TSHZ1//HMG20A//CEBPB//ZMYND11//ARX//DNMT3B//EGR2//ERG//ESR1//EZH1//BAHD1//FLI1//CIC//FMR1//NR5A2//GATA6//ATAD2//HMGB3//AR//JARID2//JUN//NEUROD1//NEUROG1//NFIA//POU4F1//PPARG//PRKAA1//TSHZ3//RAD21//REST//RNF2//GMNC//ZEB1//TFAP2A//DEK//BRD3//TCF7L1//URI1//KAT2B// |
| GO:0001047 | core promoter binding | Molecular function | 20 | 142 | 937 | 17548 | 2.63772603604552 | 6.66235963013266e-05 | 0.00278529615504901 | 4.17637192805933 | 0.0213447171824973 | CEBPB//GADD45A//H2AFZ//SOX11//TFAP2A//ESR1//PPARG//REST//SOX4//KLF10//NR4A3//CLOCK//IRF9//E2F1//E2F3//NPAS2//TFAP2C//HMGA2//KLF4//ST18// |
| GO:0008373 | sialyltransferase activity | Molecular function | 7 | 21 | 937 | 17548 | 6.24261828530772 | 7.26900523064688e-05 | 0.00294394711841199 | 4.13852501863213 | 0.00747065101387407 | ST6GALNAC3//ST6GALNAC6//ST8SIA5//ST8SIA3//ST6GAL2//ST3GAL3//ST3GAL5// |
| GO:0019900 | kinase binding | Molecular function | 64 | 739 | 937 | 17548 | 1.62189812013408 | 9.06297748843114e-05 | 0.00355927843182023 | 4.04272909875165 | 0.0683030949839915 | ITGAV//MARCKS//PLEK//UGT1A10//UGT1A7//SRSF2//YWHAG//ADAM9//SQSTM1//DUSP10//BCL2L11//ADAM10//CDKN1A//MAP3K2//OSR1//SPRED1//DCX//E2F1//ESR1//CCNY//MAPRE3//GATA6//GRB2//RHOC//RND3//MAP3K5//MSN//PPP1R12A//NEFH//NSF//ERRFI1//PPP1R12C//ZBTB4//RHEB//STAT3//NR4A3//CALM2//KAT2B//CCND2//CCNE2//CAMK2N2//BAG5//TOB1//NRG1//NCK1//SOCS5//EPHA2//EPHA4//EPHA5//EPHA7//KDR//MET//MAPK1//MAP2K4//TGFBR2//PRKAR2B//APPL1//DUSP2//CRK//GJA1//PTEN//CEBPB//GADD45A//BTG1// |
| GO:0017124 | SH3 domain binding | Molecular function | 18 | 125 | 937 | 17548 | 2.69681109925294 | 0.000113715042954286 | 0.00433454987261043 | 3.94418208025597 | 0.0192102454642476 | ADAM10//KHDRBS3//CRK//EPS15//CD2AP//GJA1//GRB2//ARHGAP1//ARHGAP6//ERRFI1//RUFY2//ENAH//PLSCR4//SOS1//CCDC6//ADAM12//ADAM9//QKI// |
| GO:0001085 | RNA polymerase II transcription factor binding | Molecular function | 19 | 141 | 937 | 17548 | 2.52361164725206 | 0.000181995900268411 | 0.00673904819279602 | 3.73993839504163 | 0.0202774813233725 | ESR1//GTF2E1//EGR2//JUN//MEF2D//NEUROD1//NHLH2//RBL2//BHLHE41//BHLHE40//TCERG1//GATA6//RBBP8//STAT3//WWP2//ZFPM2//AR//TEAD1//KLF4// |
| GO:0016740 | transferase activity | Molecular function | 168 | 2437 | 937 | 17548 | 1.29104621083098 | 0.000218661847252286 | 0.0078718265010823 | 3.66022698730195 | 0.179295624332978 | PIKFYVE//ZFYVE21//CHST7//CHST1//ST6GALNAC3//ST6GALNAC6//PTGES3//ST8SIA5//ST8SIA3//WDFY3//ST6GAL2//ST3GAL3//ST3GAL5//LCLAT1//PFKP//DNMT3B//REV3L//POLR3G//DGKH//SPHK2//DGKE//GNPTG//UGP2//BCAT2//CMPK1//CDS1//GFPT2//GSTA2//MPP1//HS2ST1//TADA1//EPC2//KAT2B//CLOCK//HPRT1//ESCO2//CMTR2//NME1//NAA30//PSAT1//HIPK3//CDK8//MAP3K2//EPHA4//MMD//PAN3//MAP3K5//MINK1//CDK17//PRKAA1//CCL2//CCL8//MAP2K4//ACVR1//PKDCC//RPS6KA5//STK17B//FASTK//OSR1//PDIK1L//DSTYK//LATS2//MKNK2//TAOK3//PRKACB//MAPK1//PAK6//FAM20C//STK33//MASTL//SQSTM1//CCNC//RPS6KA4//ULK2//SLK//TGFBR2//HMGA2//CDK6//CDKN1A//CDK19//BMPR2//EPHA2//EPHA5//EPHA7//KDR//MET//EREG//NRG1//JAK1//GRB2//PCMTD1//SPTSSA//TOPORS//WWP2//KLHL2//HECTD2//RNF38//ZNRF2//FBXL5//KLHL3//MYLIP//RNF141//RLIM//UBE2W//FBXW7//KLHL14//MARCH4//RNF2//RNF4//SKP1//KLHL15//FBXO30//KBTBD8//CDC23//LONRF1//DSEL//HS3ST3A1//HS3ST1//PIGA//UGT1A10//UGT1A8//UGT1A7//UGT1A6//UGT1A5//UGT1A9//UGT1A4//UGT1A1//UGT1A3//PTAR1//XYLT2//B3GNT5//B3GALT2//FUT9//HS3ST5//ELOVL4//ELOVL7//PIK3CB//GAB1//PIK3R3//ZDHHC5//ZDHHC1//EZH1//PRDM16//DPY30//KMT2B//ATG16L1//PIAS4//UBE2I//CCDC126//NAGK//KDELC1//ESR1//CASD1//TRIM3//RNF145//RNF180//MDM4//ANKIB1//HECW2//AKTIP//UBE2Z//RNF128//LONRF3//UBE3B//SOSTDC1//EGR2//MYCBP2//MARCH7// |
| GO:0030234 | enzyme regulator activity | Molecular function | 82 | 1041 | 937 | 17548 | 1.47520086281047 | 0.000243202145290121 | 0.00849487753822064 | 3.61403259846364 | 0.0875133404482391 | BAG5//DNAJB4//FRY//PPP1R12A//UGT1A8//UGT1A7//UGT1A1//PPP1R12C//CDKN1A//FLRT1//NCK1//TAOK3//ASPN//DUS2//SOCS5//KAT2B//PRKAR2B//PPP1R37//URI1//RECK//SERPINI1//SPOCK1//IQGAP2//CPEB2//IPO5//SLIT2//FAM13A//DLC1//CDC42EP3//GNB5//ARAP2//TAGAP//TBC1D20//RAB3GAP1//TBC1D12//APPL1//RGS17//GNAQ//AGFG1//AGFG2//JUN//ARHGAP1//ARHGAP6//ERRFI1//ELMOD1//RGS3//SOS1//EVI5//TBC1D17//TRIP10//ARHGAP12//USP6NL//GIT2//CTSA//YWHAG//CAMK2N2//CCL8//PLAA//NSMAF//PCOLCE2//CCNY//CCNC//CCNE2//SBF2//IL2//LAMTOR3//LRP6//ANP32E//UBXN2B//PPP4R2//PPP1R15B//ESR1//SPRED1//NRG1//EFNA5//WASL//KLF4//FNIP1//CALM2//PIK3R3//FBXW7//MTMR9// |
| GO:0030371 | translation repressor activity | Molecular function | 7 | 25 | 937 | 17548 | 5.24379935965848 | 0.000249078199423136 | 0.00849487753822064 | 3.60366428243764 | 0.00747065101387407 | CPEB2//CPEB3//CPEB1//CPEB4//FMR1//NANOS1//PAIP2// |
| GO:0003705 | transcription factor activity, RNA polymerase II distal enhancer sequence-specific binding | Molecular function | 15 | 101 | 937 | 17548 | 2.78136458256285 | 0.000293126237787662 | 0.00974081036340538 | 3.53294530603648 | 0.016008537886873 | MEF2D//MEOX2//PBX3//POU4F1//ARX//CEBPB//FOXF1//FOXF2//FOXC1//NR5A2//GATA6//JUN//SOX17//BHLHE41//BHLHE40// |
| GO:0043167 | ion binding | Molecular function | 378 | 6159 | 937 | 17548 | 1.14939586548773 | 0.000347415272570866 | 0.0112562548312961 | 3.45915109363947 | 0.4034151547492 | ACOT7//ACBD3//ACBD5//HPRT1//IDH1//MAP3K5//NME1//PRKACB//PTEN//RHEB//DCTPP1//RPS6KA4//RPS6KA5//OSBPL5//FCHO2//SYT6//UGT1A8//UGT1A7//UGT1A9//UGT1A1//UGT1A3//EGLN3//FBXL5//EGLN1//MSMO1//SCD5//CETN1//DSC2//EPS15//SULF1//NPNT//SLC24A2//CDH20//DLL1//MICU3//LDLR//MAN1A1//ASPH//PKD2//ASPN//PPP3R1//PRRG1//FSTL5//FAM20C//PLSCR4//SLC24A3//STIM2//SMOC1//SMOC2//SPOCK1//VLDLR//LRP8//PRRG4//CALM2//PCDH11Y//CALN1//MCFD2//SLIT2//CD69//EDEM1//EFCAB14//HIPK3//CDK6//CDK8//MAP3K2//ADCY3//FASTK//CLCN5//CLCN6//PDIK1L//DGKH//DDX6//DYNC1LI2//EPHA2//PIKFYVE//EPHA4//EPHA5//EPHA7//ACSL1//CDK19//PAN3//DSTYK//LATS2//MKNK2//ATAD2//JAK1//KCNJ10//KDR//KIF5A//MET//MYO1B//NSF//P2RY1//MINK1//CDK17//TAOK3//CMPK1//PFKP//PIK3CB//UBE2W//NAGK//PRKAA1//MAPK1//SPHK2//PAK6//RIMKLB//TWF1//KIF13A//MAP2K4//UBE2Z//BMPR2//STK33//HSPA13//ACTC1//TGFBR2//UBE2I//FYCO1//UBA5//MASTL//DGKE//RTCA//RUNX3//ACVR1//BTAF1//CCT6A//PKDCC//STK17B//ITM2B//KIF23//ULK2//SLK//RAB12//RAB21//RAB38//ARL5A//RAB30//GLUD2//GNAQ//NKIRAS1//RHOC//RND3//SAR1B//RAB9B//DNAJC27//RASD1//RAB23//ARL15//RAB22A//RAP2C//RRAGD//RAP1B//RASL11B//RAB28//HAPLN1//NUP35//F3//LPAR3//NR5A2//ARFIP1//PSD//WDFY3//ZFYVE9//AMER2//PFN2//SESTD1//IQGAP2//ARAP2//PLEKHB2//PCOLCE2//CCL8//VEGFB//SEC23A//TRIM3//ZMYND11//RNF145//ESR1//ZCCHC24//NAPEPLD//KDM2A//MYT1L//ZFPM2//MORC3//TES//GATA6//GLRA1//ZADH2//NANOS1//AR//MDM4//NR3C2//MMP2//NR4A2//PIAS4//ZFR//ZFAND6//PPARG//ZNFX1//MARCH4//RARB//RNF2//RNF4//CLIP1//ZMAT3//MARCH7//ZEB1//THRA//ZNF2//ZNF3//NR4A3//FBXO30//USP45//ZCCHC3//KLF7//SQSTM1//KLF4//ZFAND4//ST18//KMT2B//USP3//MTRR//GLRB//MIEF1//DCP2//NPEPL1//PDE10A//RAPGEF4//PRKAR2B//HCN2//KCNA4//CHMP3//PLEKHF2//ST8SIA3//SNX5//PXK//RUFY2//PLEKHA1//AIDA//SNX2//SBF2//DRD1//LPAR1//PLEK//TSHZ1//ADAM10//TOPORS//CDKN1A//KLF2//ZBTB18//ZNF268//KLF12//FGD4//COL1A2//OSR1//KLF6//ZNF597//PPM1L//RNF38//FGD5//ESCO2//ZNF782//ZNF800//GLIS3//DNMT3B//ZNF367//EGR2//ZFC3H1//ZBTB9//ZNRF2//FHL2//PPM1E//DZIP1//ENPP4//MYCBP2//CNOT6L//STEAP2//LHX6//GNS//RNF180//MYLIP//GTF2E1//CNOT7//HIVEP2//AGFG1//AGFG2//ZBTB41//IMPDH1//ITGA6//ITGA5//ITGAV//ZNF568//YPEL2//LHX1//ZBTB34//MAN2A1//MBNL1//MLLT6//LHX8//ACO1//NPAS2//RNF141//RLIM//KLF3//ZBTB7A//LIMA1//PON2//ANKIB1//RNF186//RSBN1//BNC2//SOBP//PPP2CA//ARMC1//ZNF532//PPP6C//TRPV6//NPLOC4//TRERF1//LMO3//SLC2A4RG//ZNF630//ZNF287//CNOT6//ZNF512B//NLN//TSHZ3//ZBTB4//ZBTB26//ENPP5//REST//REV3L//RRM2//DMRTA1//PRDM16//ARHGEF28//CPEB1//USP46//BCL11B//SLC6A1//CAPRIN2//TGFB1I1//THOP1//KLF10//UGP2//ZNF131//ZNF217//ZNF655//ZFYVE21//RNF128//ADIPOR2//LONRF3//CYBRD1//CPEB4//ADAM12//DDHD1//SNN//HDHD2//PCGF5//ZIC5//UNK//ZC3H12C//ADAM9//LMLN//LONRF1//ZNF804A//ZBTB47//PPM1F//PHF14//GIT2//HS3ST5//ACOX1//DUS2//PLEKHA3//UXS1//TRPC3//SESN3//SESN1//CRY2//DENND1B// |
| GO:0030695 | GTPase regulator activity | Molecular function | 32 | 316 | 937 | 17548 | 1.89649163097956 | 0.00039571077907474 | 0.0125083212117284 | 3.40262211948651 | 0.0341515474919957 | IQGAP2//CPEB2//IPO5//SLIT2//FAM13A//DLC1//CDC42EP3//GNB5//ARAP2//TAGAP//TBC1D20//RAB3GAP1//TBC1D12//APPL1//RGS17//GNAQ//AGFG1//AGFG2//JUN//ARHGAP1//ARHGAP6//ERRFI1//ELMOD1//RGS3//SOS1//EVI5//TBC1D17//TRIP10//ARHGAP12//USP6NL//GIT2//WASL// |
| GO:0046875 | ephrin receptor binding | Molecular function | 7 | 27 | 937 | 17548 | 4.85536977746156 | 0.00041902612352412 | 0.0129299489544586 | 3.37775890078379 | 0.00747065101387407 | CRK//EFNA1//EFNA5//EPHA4//EPHA7//GRB2//NCK1// |
| GO:0060589 | nucleoside-triphosphatase regulator activity | Molecular function | 35 | 359 | 937 | 17548 | 1.82583543163596 | 0.000441300852394836 | 0.0133006024349699 | 3.35526523369032 | 0.0373532550693703 | BAG5//DNAJB4//IQGAP2//CPEB2//IPO5//SLIT2//FAM13A//DLC1//CDC42EP3//GNB5//ARAP2//TAGAP//TBC1D20//RAB3GAP1//TBC1D12//APPL1//RGS17//GNAQ//AGFG1//AGFG2//JUN//ARHGAP1//ARHGAP6//ERRFI1//ELMOD1//RGS3//SOS1//EVI5//TBC1D17//TRIP10//ARHGAP12//USP6NL//GIT2//WASL//FNIP1// |
| GO:0033613 | activating transcription factor binding | Molecular function | 12 | 73 | 937 | 17548 | 3.07855148316545 | 0.000452051575535084 | 0.013314973679397 | 3.34481201277166 | 0.0128068303094984 | EGR2//JUN//MEF2D//NEUROD1//NHLH2//RBL2//BHLHE41//BHLHE40//PPARG//PRDM16//WFS1//HAND2// |
| GO:0061659 | ubiquitin-like protein ligase activity | Molecular function | 25 | 229 | 937 | 17548 | 2.04452563929292 | 0.000556219666065433 | 0.0158157158393611 | 3.25475366000133 | 0.0266808964781217 | TOPORS//TRIM3//WWP2//HECTD2//RNF38//RNF145//ZNRF2//RNF180//MYLIP//MDM4//RLIM//ANKIB1//UBE2W//HECW2//RNF2//RNF4//AKTIP//SKP1//UBE2Z//RNF128//LONRF3//FBXO30//UBE3B//LONRF1//PIAS4// |
| GO:0001076 | transcription factor activity, RNA polymerase II transcription factor binding | Molecular function | 22 | 191 | 937 | 17548 | 2.15713511429481 | 0.000566945023018913 | 0.0158157158393611 | 3.24645905284805 | 0.0234791889007471 | TCERG1//TRERF1//ZFPM2//NEUROD1//POU3F2//SOX4//SOX11//RBBP8//URI1//MYB//MYBL1//WWP2//JUN//KLF4//CLOCK//CNOT7//BHLHE41//BHLHE40//GTF2E1//PPARG//RARB//THRA// |
| GO:0005041 | low-density lipoprotein particle receptor activity | Molecular function | 5 | 14 | 937 | 17548 | 6.68851959140113 | 0.00057356376886572 | 0.0158157158393611 | 3.2414182901831 | 0.00533617929562433 | LRP12//LDLR//LRP6//VLDLR//LRP8// |
| GO:0070888 | E-box binding | Molecular function | 8 | 37 | 937 | 17548 | 4.0492659147942 | 0.000620073424317181 | 0.0167419824565639 | 3.20755688165052 | 0.00853788687299893 | NEUROD1//NEUROG1//ZEB1//TCF21//BHLHE41//BHLHE40//HAND2//CLOCK// |
| GO:0003714 | transcription corepressor activity | Molecular function | 25 | 232 | 937 | 17548 | 2.01808780775034 | 0.000673862679836134 | 0.0178229802666863 | 3.17142859525323 | 0.0266808964781217 | TCERG1//RBBP8//URI1//TOB1//TOB2//ZMYND11//KLF12//DNMT3B//DR1//ELK3//ZFPM2//HSBP1//LHX1//NPAT//RLIM//PIAS4//POU4F1//SRSF2//ZEB1//TGIF1//TSG101//BHLHE41//TBL1XR1//BHLHE40//HAND1// |
| GO:0042802 | identical protein binding | Molecular function | 118 | 1661 | 937 | 17548 | 1.33045567308786 | 0.000725149571522896 | 0.0187958768938735 | 3.13957240530851 | 0.125933831376734 | ADAM10//CALCOCO2//CEBPB//ACOT7//DYNLL2//SYT6//GADD45A//FMR1//CADM2//CDH20//GRHL1//HPRT1//IDH1//INHBB//JUN//RBM44//FIBIN//LRP6//MEF2D//MAP3K5//NEUROG1//NR4A2//CHMP3//PKD2//PLEK//UGT1A10//UGT1A8//UGT1A7//UGT1A6//UGT1A9//UGT1A4//UGT1A1//UGT1A3//DSCAML1//ZBTB4//CLIP1//DMRTA1//BNIP3//SNX2//STAT3//TFAP2A//TGFB2//TSG101//VEGFB//BHLHE41//NR4A3//UXS1//FXR1//SBF2//GNPTG//DPY30//BHLHE40//SQSTM1//ACVR1//NOG//SLIT2//HAND1//HAND2//CD69//SLK//HMG20A//KHDRBS3//TCERG1//KLHL2//FCHO2//CLCN5//COL1A2//CRKL//APCDD1//DPYSL2//EPHA4//EPS15//ESR1//ETS1//NAPEPLD//FHL2//MAPRE3//ARL6IP1//APPL1//GJB2//RASSF3//RGMB//GRB2//SCHIP1//HSBP1//KDR//LDLR//MARCKS//MET//NME1//CLDN11//ST8SIA3//RAB9B//IER5//PON2//MIEF1//POU3F2//PPARG//ATG16L1//FBXW7//MAPK1//PTEN//ZBTB26//PTPRG//MID1IP1//RBBP8//RNF4//HCN2//ATXN1//STAT4//TNF//TTR//UGP2//YWHAG//ZNF3//DCTPP1//ADIPOR2//TRIP10// |
| GO:0045125 | bioactive lipid receptor activity | Molecular function | 5 | 15 | 937 | 17548 | 6.24261828530772 | 0.000822710762162321 | 0.0206717866107411 | 3.08475282150122 | 0.00533617929562433 | S1PR1//GPR6//SPHK2//LPAR1//LPAR3// |
| GO:0001134 | transcription factor activity, transcription factor recruiting | Molecular function | 7 | 30 | 937 | 17548 | 4.3698327997154 | 0.000835050118730419 | 0.0206717866107411 | 3.07828745788783 | 0.00747065101387407 | KLF4//PPARG//RARB//THRA//MYB//MYBL1//TEAD1// |
| GO:0051020 | GTPase binding | Molecular function | 55 | 665 | 937 | 17548 | 1.54892032642973 | 0.000845373989482468 | 0.0206717866107411 | 3.07295111870006 | 0.0586979722518677 | RAPGEF4//TAGAP//FGD4//FGD5//RAB3GAP1//DOCK9//ARHGEF4//RALGPS2//ARHGEF28//SOS1//SOS2//DOCK5//LAMTOR3//FNIP1//PSD//RANBP9//ADRB1//EREG//RASGEF1A//GRB2//NRG1//IL2//JAK1//MET//RGL1//IPO5//SPHK2//IQGAP2//CDC42EP3//DENND5B//DENND1B//TRAPPC8//SBF2//TBC1D20//TBC1D12//ARHGAP1//NSF//EVI5//TBC1D17//USP6NL//EXOC5//EPS8//ARAP2//APPL1//AGFG1//AGFG2//PEX5L//ERRFI1//GIT2//PAK6//GOLGA4//POU4F1//ATG16L1//RRAGD//BNIP3// |
| GO:0061630 | ubiquitin protein ligase activity | Molecular function | 24 | 224 | 937 | 17548 | 2.00655587742034 | 0.000926573167236498 | 0.0218383433021305 | 3.03312028074613 | 0.0256136606189968 | TOPORS//TRIM3//WWP2//HECTD2//RNF38//RNF145//ZNRF2//RNF180//MYLIP//MDM4//RLIM//ANKIB1//UBE2W//HECW2//RNF2//RNF4//AKTIP//SKP1//UBE2Z//RNF128//LONRF3//FBXO30//UBE3B//LONRF1// |
| GO:0019904 | protein domain specific binding | Molecular function | 57 | 698 | 937 | 17548 | 1.52935204410834 | 0.000926781544457699 | 0.0218383433021305 | 3.03302262315568 | 0.0608324439701174 | ADAM10//KHDRBS3//CRK//EPS15//CD2AP//GJA1//GRB2//ARHGAP1//ARHGAP6//ERRFI1//RUFY2//ENAH//PLSCR4//SOS1//CCDC6//ADAM12//ADAM9//QKI//ADRB1//LPAR1//GRIA1//NSF//ACOX1//PTEN//PLEKHA1//HMGA2//DLC1//SQSTM1//EPHA4//PKD2//UBE2I//PPARG//NFE2//NDFIP2//PMEPA1//STRN//LBR//AR//RNF2//EGR2//HOXA3//JUN//CCT6A//CALM2//DDX6//ARFIP1//MAP3K5//NCK1//ARHGEF4//PPP3R1//PRKAR2B//AIDA//SKP1//THRA//YWHAG//BHLHE40//ZFYVE9// |
| GO:0004879 | nuclear receptor activity | Molecular function | 9 | 49 | 937 | 17548 | 3.43981007557772 | 0.00101263728191208 | 0.0228102747725556 | 2.99454608738703 | 0.0096051227321238 | RARB//AR//THRA//NR4A3//ESR1//NR5A2//NR4A2//PPARG//STAT3// |
| GO:0098531 | transcription factor activity, direct ligand regulated sequence-specific DNA binding | Molecular function | 9 | 49 | 937 | 17548 | 3.43981007557772 | 0.00101263728191208 | 0.0228102747725556 | 2.99454608738703 | 0.0096051227321238 | RARB//NR5A2//AR//NR4A2//PPARG//STAT3//THRA//NR4A3//ESR1// |
| GO:0003707 | steroid hormone receptor activity | Molecular function | 10 | 59 | 937 | 17548 | 3.1742126874446 | 0.00103843071881233 | 0.0228102747725556 | 2.98362247307322 | 0.0106723585912487 | AR//ESR1//PGRMC2//NR5A2//NR3C2//NR4A2//PPARG//RARB//THRA//NR4A3// |
| GO:0019003 | GDP binding | Molecular function | 10 | 59 | 937 | 17548 | 3.1742126874446 | 0.00103843071881233 | 0.0228102747725556 | 2.98362247307322 | 0.0106723585912487 | RAB12//RAB21//RAB9B//MIEF1//RAB22A//RAP2C//RRAGD//RAP1B//RHEB//RAB28// |
| GO:0005080 | protein kinase C binding | Molecular function | 9 | 50 | 937 | 17548 | 3.37101387406617 | 0.00117738163300756 | 0.0254314432729633 | 2.92908274340552 | 0.0096051227321238 | ITGAV//MARCKS//PLEK//UGT1A10//UGT1A7//SRSF2//YWHAG//ADAM9//SQSTM1// |
| GO:0030228 | lipoprotein particle receptor activity | Molecular function | 5 | 17 | 937 | 17548 | 5.50819260468328 | 0.00155051048766284 | 0.0329419933116564 | 2.80952529184607 | 0.00533617929562433 | LRP12//LDLR//LRP6//VLDLR//LRP8// |
| GO:0016773 | phosphotransferase activity, alcohol group as acceptor | Molecular function | 61 | 778 | 937 | 17548 | 1.46837936531017 | 0.00167087500269894 | 0.0349266774757714 | 2.77705603823862 | 0.0651013874066169 | PIKFYVE//ZFYVE21//PFKP//DGKH//SPHK2//DGKE//HIPK3//CDK8//MAP3K2//EPHA4//MMD//PAN3//MAP3K5//MINK1//CDK17//PRKAA1//CCL2//CCL8//MAP2K4//ACVR1//PKDCC//RPS6KA5//STK17B//FASTK//OSR1//PDIK1L//DSTYK//LATS2//MKNK2//TAOK3//PRKACB//MAPK1//PAK6//FAM20C//STK33//MASTL//SQSTM1//CCNC//RPS6KA4//ULK2//SLK//TGFBR2//HMGA2//CDK6//CDKN1A//CDK19//BMPR2//EPHA2//EPHA5//EPHA7//KDR//MET//EREG//NRG1//JAK1//GRB2//GAB1//PIK3CB//PIK3R3//ESR1//SOSTDC1// |
| GO:0046332 | SMAD binding | Molecular function | 11 | 74 | 937 | 17548 | 2.78387031642101 | 0.00180980581464232 | 0.0370518991074314 | 2.74236802079572 | 0.0117395944503735 | TGIF1//TGFB1I1//JUN//PMEPA1//LDLRAD4//TOB1//COL1A2//PRDM16//TGFBR2//HMGA2//ACVR1// |
| GO:0019207 | kinase regulator activity | Molecular function | 23 | 222 | 937 | 17548 | 1.94027325083889 | 0.00182972341271266 | 0.0370518991074314 | 2.73761455475651 | 0.0245464247598719 | CDKN1A//FLRT1//NCK1//TAOK3//ASPN//DUS2//SOCS5//KAT2B//PRKAR2B//YWHAG//CAMK2N2//CCNY//CCNC//CCNE2//IL2//LAMTOR3//LRP6//SPRED1//NRG1//EFNA5//KLF4//CALM2//PIK3R3// |
| GO:0035091 | phosphatidylinositol binding | Molecular function | 24 | 237 | 937 | 17548 | 1.89649163097956 | 0.00200337696321699 | 0.0399442545281418 | 2.69823732447497 | 0.0256136606189968 | WDFY3//ZFYVE9//FCHO2//AMER2//MYO1B//PFN2//TWF1//SESTD1//IQGAP2//ARAP2//PLEKHB2//PLEKHF2//PLEK//PLEKHA1//OSBPL5//ARFIP1//PLEKHA3//DENND1B//SNX5//PXK//RUFY2//AIDA//SNX2//SBF2// |
| GO:0001972 | retinoic acid binding | Molecular function | 5 | 18 | 937 | 17548 | 5.2021819044231 | 0.00205334885552541 | 0.0403203047994081 | 2.68753725951455 | 0.00533617929562433 | UGT1A8//UGT1A7//UGT1A9//UGT1A1//UGT1A3// |
| GO:0016301 | kinase activity | Molecular function | 65 | 850 | 937 | 17548 | 1.43213007721765 | 0.00219626106294241 | 0.041422291826237 | 2.65831603787559 | 0.0693703308431163 | PIKFYVE//ZFYVE21//PFKP//DGKH//SPHK2//DGKE//CMPK1//MPP1//NME1//HIPK3//CDK8//MAP3K2//EPHA4//MMD//PAN3//MAP3K5//MINK1//CDK17//PRKAA1//CCL2//CCL8//MAP2K4//ACVR1//PKDCC//RPS6KA5//STK17B//FASTK//OSR1//PDIK1L//DSTYK//LATS2//MKNK2//TAOK3//PRKACB//MAPK1//PAK6//FAM20C//STK33//MASTL//SQSTM1//CCNC//RPS6KA4//ULK2//SLK//TGFBR2//HMGA2//CDK6//CDKN1A//CDK19//BMPR2//EPHA2//EPHA5//EPHA7//KDR//MET//EREG//NRG1//JAK1//GRB2//GAB1//PIK3CB//PIK3R3//NAGK//ESR1//SOSTDC1// |
| GO:0001046 | core promoter sequence-specific DNA binding | Molecular function | 12 | 87 | 937 | 17548 | 2.58315239392044 | 0.00220314469278536 | 0.041422291826237 | 2.65695697937456 | 0.0128068303094984 | CEBPB//GADD45A//H2AFZ//SOX11//TFAP2A//ESR1//PPARG//REST//SOX4//KLF10//NR4A3//CLOCK// |
| GO:0098772 | molecular function regulator | Molecular function | 126 | 1848 | 937 | 17548 | 1.27689919472203 | 0.00220535350000799 | 0.041422291826237 | 2.65652178678665 | 0.134471718249733 | BAG5//DNAJB4//FRY//PPP1R12A//UGT1A8//UGT1A7//UGT1A1//PPP1R12C//CDKN1A//FLRT1//NCK1//TAOK3//ASPN//DUS2//SOCS5//KAT2B//PRKAR2B//PPP1R37//URI1//RECK//SERPINI1//SPOCK1//RAPGEF4//TAGAP//FGD4//FGD5//RAB3GAP1//DOCK9//ARHGEF4//RALGPS2//ARHGEF28//SOS1//SOS2//DOCK5//LAMTOR3//FNIP1//PSD//RANBP9//ADRB1//EREG//RASGEF1A//GRB2//NRG1//IL2//JAK1//MET//IQGAP2//CPEB2//IPO5//SLIT2//FAM13A//DLC1//CDC42EP3//GNB5//ARAP2//TBC1D20//TBC1D12//APPL1//RGS17//GNAQ//AGFG1//AGFG2//JUN//ARHGAP1//ARHGAP6//ERRFI1//ELMOD1//RGS3//EVI5//TBC1D17//TRIP10//ARHGAP12//USP6NL//GIT2//GDF11//EDN1//IFNA1//INHBB//OSM//BMP3//TGFB2//TNF//WNT7A//IGF1//PTHLH//TTR//STIM2//CCL2//CCL8//CXCL14//CTSA//NTF3//OGN//VEGFB//RGL1//YWHAG//CAMK2N2//PLAA//NSMAF//PCOLCE2//CCNY//CCNC//CCNE2//DENND5B//DENND1B//TRAPPC8//SBF2//LRP6//ANP32E//CALM2//UBXN2B//PPP4R2//PPP1R15B//MTMR9//ESR1//SPRED1//EFNA5//LYPD6//EPS8//WASL//KLF4//SEMA4B//EPHA7//PIK3R3//DKK3//FBXW7// |
| GO:0019210 | kinase inhibitor activity | Molecular function | 13 | 99 | 937 | 17548 | 2.4592132639091 | 0.00230447105460444 | 0.0423655851332227 | 2.63742874247742 | 0.0138740661686233 | CDKN1A//FLRT1//NCK1//TAOK3//ASPN//DUS2//SOCS5//KAT2B//PRKAR2B//YWHAG//CAMK2N2//SPRED1//LRP6// |
| GO:0005085 | guanyl-nucleotide exchange factor activity | Molecular function | 30 | 323 | 937 | 17548 | 1.73942924358419 | 0.00232095412381081 | 0.0423655851332227 | 2.63433344376326 | 0.032017075773746 | PSD//RANBP9//RAPGEF4//ADRB1//EREG//RASGEF1A//GRB2//NRG1//IL2//JAK1//MET//SOS1//FGD4//FGD5//ARHGEF4//ARHGEF28//SOS2//RGL1//DENND5B//DENND1B//TRAPPC8//RAB3GAP1//SBF2//EPS8//TAGAP//DOCK9//RALGPS2//DOCK5//LAMTOR3//FNIP1// |
| GO:0046982 | protein heterodimerization activity | Molecular function | 42 | 500 | 937 | 17548 | 1.57313980789755 | 0.00250559670271783 | 0.0451007406489209 | 2.60108883113891 | 0.0448239060832444 | BCL2L11//HMG20A//CEBPB//DYNLL2//ADRB1//GADD45A//DR1//FMR1//SNX5//H2AFZ//JUN//MEF2D//NEUROD1//NR4A2//P2RY1//UGT1A10//UGT1A8//UGT1A7//UGT1A6//UGT1A9//UGT1A4//UGT1A1//UGT1A3//PPARG//PPP2CA//RRAGD//SOX17//BNIP3//SNX2//SOS1//SOS2//SOX4//TEAD1//TGFB2//TTR//VEGFB//BHLHE41//ADIPOR2//FXR1//BHLHE40//HAND1//HAND2// |
| GO:0008138 | protein tyrosine/serine/threonine phosphatase activity | Molecular function | 8 | 46 | 937 | 17548 | 3.25701823581272 | 0.00274140742211874 | 0.0486693701241902 | 2.56202641574506 | 0.00853788687299893 | DUSP10//DUSP1//DUSP2//DUSP5//PTPDC1//PTEN//PTP4A1//CDC14A// |
| GO:0005096 | GTPase activator activity | Molecular function | 27 | 286 | 937 | 17548 | 1.76801426961512 | 0.00298656178417695 | 0.0516691252492255 | 2.52482849640341 | 0.0288153681963714 | FAM13A//DLC1//CDC42EP3//GNB5//ARAP2//TAGAP//TBC1D20//RAB3GAP1//TBC1D12//APPL1//RGS17//GNAQ//AGFG1//AGFG2//JUN//ARHGAP1//ARHGAP6//ERRFI1//ELMOD1//RGS3//SOS1//EVI5//TBC1D17//TRIP10//ARHGAP12//USP6NL//GIT2// |
| GO:0004674 | protein serine/threonine kinase activity | Molecular function | 42 | 505 | 937 | 17548 | 1.55756416623519 | 0.00300119341561019 | 0.0516691252492255 | 2.52270601502985 | 0.0448239060832444 | TGFBR2//ACVR1//HMGA2//PRKAA1//MKNK2//PRKACB//CDK6//CDK8//CDKN1A//CDK19//CDK17//BMPR2//MAPK1//MAP3K2//EPHA2//EPHA4//EPHA5//EPHA7//KDR//MAP3K5//MET//TAOK3//RPS6KA4//MINK1//FASTK//SOSTDC1//HIPK3//OSR1//PDIK1L//DSTYK//LATS2//PAK6//FAM20C//MAP2K4//STK33//MASTL//SQSTM1//CCNC//RPS6KA5//STK17B//ULK2//SLK// |
| GO:0005070 | SH3/SH2 adaptor activity | Molecular function | 9 | 57 | 937 | 17548 | 2.95702971409313 | 0.00302997956708421 | 0.0516691252492255 | 2.51856030018829 | 0.0096051227321238 | TOB1//CRK//CRKL//EPS8//GAB1//GRB2//ARHGAP1//ARHGAP6//NCK1// |
| GO:0004672 | protein kinase activity | Molecular function | 51 | 644 | 937 | 17548 | 1.48310651809329 | 0.00317820546071392 | 0.0534929126894187 | 2.49781803048609 | 0.0544290288153682 | HIPK3//CDK8//MAP3K2//FASTK//OSR1//PDIK1L//DSTYK//LATS2//MKNK2//MAP3K5//MINK1//CDK17//TAOK3//PRKAA1//PRKACB//MAPK1//PAK6//FAM20C//MAP2K4//STK33//MASTL//SQSTM1//CCNC//RPS6KA4//ACVR1//RPS6KA5//STK17B//ULK2//SLK//TGFBR2//HMGA2//CDK6//CDKN1A//CDK19//BMPR2//EPHA2//EPHA4//EPHA5//EPHA7//KDR//MET//EREG//NRG1//JAK1//GRB2//PKDCC//SOSTDC1//MMD//PAN3//CCL2//CCL8// |
| GO:0043169 | cation binding | Molecular function | 261 | 4225 | 937 | 17548 | 1.15691600411738 | 0.0034206164043815 | 0.0568348571804926 | 2.4658956258445 | 0.27854855923159 | HPRT1//IDH1//MAP3K5//NME1//PRKACB//PTEN//RHEB//DCTPP1//RPS6KA4//RPS6KA5//EGLN3//FBXL5//EGLN1//MSMO1//SCD5//CETN1//SYT6//DSC2//EPS15//SULF1//NPNT//SLC24A2//CDH20//DLL1//MICU3//LDLR//MAN1A1//ASPH//PKD2//ASPN//PPP3R1//PRRG1//FSTL5//FAM20C//PLSCR4//SLC24A3//STIM2//SMOC1//SMOC2//SPOCK1//VLDLR//LRP8//PRRG4//CALM2//PCDH11Y//CALN1//MCFD2//SLIT2//CD69//EDEM1//EFCAB14//SEC23A//TRIM3//ZMYND11//RNF145//PIKFYVE//ESR1//ZCCHC24//NAPEPLD//KDM2A//MYT1L//ZFPM2//MORC3//NR5A2//TES//GATA6//GLRA1//ZADH2//NANOS1//AR//MDM4//NR3C2//MMP2//NR4A2//PIAS4//ZFR//ZFAND6//PPARG//ZNFX1//MARCH4//RARB//RNF2//RNF4//CLIP1//ZMAT3//MARCH7//ZEB1//THRA//ZNF2//ZNF3//NR4A3//FBXO30//USP45//ZCCHC3//KLF7//SQSTM1//KLF4//ZFAND4//ST18//KMT2B//USP3//GLRB//DCP2//NPEPL1//KCNA4//CHMP3//DRD1//TSHZ1//ADAM10//TOPORS//CDKN1A//KLF2//ZBTB18//MAP3K2//ZNF268//PDE10A//ADCY3//KLF12//ARAP2//FGD4//COL1A2//OSR1//KLF6//ZNF597//PPM1L//RNF38//FGD5//ESCO2//ZNF782//DGKH//ZNF800//GLIS3//DNMT3B//ZNF367//EGR2//ZFC3H1//ZBTB9//ZNRF2//FHL2//PPM1E//DZIP1//ENPP4//WDFY3//MYCBP2//CNOT6L//PAN3//STEAP2//LHX6//LATS2//GNAQ//GNS//RNF180//MKNK2//MYLIP//GTF2E1//CNOT7//HIVEP2//AGFG1//AGFG2//ZBTB41//IMPDH1//ITGA6//ITGA5//ITGAV//JAK1//ZNF568//YPEL2//LHX1//ZBTB34//MAN2A1//MBNL1//MLLT6//LHX8//ACO1//NPAS2//NSF//RNF141//SAR1B//RLIM//KLF3//ZBTB7A//LIMA1//PFKP//PON2//ANKIB1//RNF186//RSBN1//BNC2//SOBP//PPP2CA//ARMC1//ZNF532//PPP6C//TRPV6//PRKAA1//NPLOC4//RUFY2//TRERF1//LMO3//SLC2A4RG//ZNF630//ZNF287//CNOT6//ZNF512B//NLN//RIMKLB//TSHZ3//ZBTB4//ZBTB26//ENPP5//REST//REV3L//RRM2//DMRTA1//PRDM16//ARHGEF28//CPEB1//USP46//BCL11B//SLC6A1//BMPR2//CAPRIN2//TGFB1I1//TGFBR2//THOP1//KLF10//UGP2//ZNF131//ZNF217//ZNF655//ZFYVE21//FYCO1//RNF128//ADIPOR2//PLEKHF2//LONRF3//UBA5//CYBRD1//CPEB4//ADAM12//DDHD1//SNN//HDHD2//PCGF5//DGKE//ZIC5//UNK//ZC3H12C//ADAM9//LMLN//ACVR1//LONRF1//ZNF804A//ZBTB47//ZFYVE9//PPM1F//PHF14//GIT2//SESN3//SESN1//GLUD2// |
| GO:1990782 | protein tyrosine kinase binding | Molecular function | 10 | 70 | 937 | 17548 | 2.67540783656045 | 0.00386776002831579 | 0.0626884994684427 | 2.41254047916279 | 0.0106723585912487 | TOB1//NRG1//NCK1//YWHAG//SQSTM1//SOCS5//CRK//EPHA4//GJA1//PTEN// |
| GO:0004535 | poly(A)-specific ribonuclease activity | Molecular function | 4 | 13 | 937 | 17548 | 5.76241687874559 | 0.00391803121677767 | 0.0626884994684427 | 2.40693210810129 | 0.00426894343649947 | CNOT6L//PAN3//CNOT7//CNOT6// |
| GO:0017017 | MAP kinase tyrosine/serine/threonine phosphatase activity | Molecular function | 4 | 13 | 937 | 17548 | 5.76241687874559 | 0.00391803121677767 | 0.0626884994684427 | 2.40693210810129 | 0.00426894343649947 | DUSP10//DUSP1//DUSP2//DUSP5// |
| GO:0046872 | metal ion binding | Molecular function | 255 | 4131 | 937 | 17548 | 1.15604042320513 | 0.00403876181218249 | 0.0635806323503383 | 2.39375175878967 | 0.272145144076841 | HPRT1//IDH1//MAP3K5//NME1//PRKACB//PTEN//RHEB//DCTPP1//RPS6KA4//RPS6KA5//EGLN3//FBXL5//EGLN1//MSMO1//SCD5//CETN1//SYT6//DSC2//EPS15//SULF1//NPNT//SLC24A2//CDH20//DLL1//MICU3//LDLR//MAN1A1//ASPH//PKD2//ASPN//PPP3R1//PRRG1//FSTL5//FAM20C//PLSCR4//SLC24A3//STIM2//SMOC1//SMOC2//SPOCK1//VLDLR//LRP8//PRRG4//CALM2//PCDH11Y//CALN1//MCFD2//SLIT2//CD69//EDEM1//EFCAB14//SEC23A//TRIM3//ZMYND11//RNF145//PIKFYVE//ESR1//ZCCHC24//NAPEPLD//KDM2A//MYT1L//ZFPM2//MORC3//NR5A2//TES//GATA6//GLRA1//ZADH2//NANOS1//AR//MDM4//NR3C2//MMP2//NR4A2//PIAS4//ZFR//ZFAND6//PPARG//ZNFX1//MARCH4//RARB//RNF2//RNF4//CLIP1//ZMAT3//MARCH7//ZEB1//THRA//ZNF2//ZNF3//NR4A3//FBXO30//USP45//ZCCHC3//KLF7//SQSTM1//KLF4//ZFAND4//ST18//KMT2B//USP3//DCP2//NPEPL1//KCNA4//TSHZ1//ADAM10//TOPORS//CDKN1A//KLF2//ZBTB18//MAP3K2//ZNF268//PDE10A//ADCY3//KLF12//ARAP2//FGD4//COL1A2//OSR1//KLF6//ZNF597//PPM1L//RNF38//FGD5//ESCO2//ZNF782//DGKH//ZNF800//GLIS3//DNMT3B//ZNF367//EGR2//ZFC3H1//ZBTB9//ZNRF2//FHL2//PPM1E//DZIP1//ENPP4//WDFY3//MYCBP2//CNOT6L//PAN3//STEAP2//LHX6//LATS2//GNAQ//GNS//RNF180//MKNK2//MYLIP//GTF2E1//CNOT7//HIVEP2//AGFG1//AGFG2//ZBTB41//IMPDH1//ITGA6//ITGA5//ITGAV//JAK1//ZNF568//YPEL2//LHX1//ZBTB34//MAN2A1//MBNL1//MLLT6//LHX8//ACO1//NPAS2//NSF//RNF141//SAR1B//RLIM//KLF3//ZBTB7A//LIMA1//PFKP//PON2//ANKIB1//RNF186//RSBN1//BNC2//SOBP//PPP2CA//ARMC1//ZNF532//PPP6C//TRPV6//PRKAA1//NPLOC4//RUFY2//TRERF1//LMO3//SLC2A4RG//ZNF630//ZNF287//CNOT6//ZNF512B//NLN//RIMKLB//TSHZ3//ZBTB4//ZBTB26//ENPP5//REST//REV3L//RRM2//DMRTA1//PRDM16//ARHGEF28//CPEB1//USP46//BCL11B//SLC6A1//BMPR2//CAPRIN2//TGFB1I1//TGFBR2//THOP1//KLF10//UGP2//ZNF131//ZNF217//ZNF655//ZFYVE21//FYCO1//RNF128//ADIPOR2//PLEKHF2//LONRF3//UBA5//CYBRD1//CPEB4//ADAM12//DDHD1//SNN//HDHD2//PCGF5//DGKE//ZIC5//UNK//ZC3H12C//ADAM9//LMLN//ACVR1//LONRF1//ZNF804A//ZBTB47//ZFYVE9//PPM1F//PHF14//GIT2// |
| GO:0001102 | RNA polymerase II activating transcription factor binding | Molecular function | 8 | 49 | 937 | 17548 | 3.05760895606909 | 0.00411582501045872 | 0.0635806323503383 | 2.38554309810001 | 0.00853788687299893 | EGR2//JUN//MEF2D//NEUROD1//NHLH2//RBL2//BHLHE41//BHLHE40// |
| GO:0008013 | beta-catenin binding | Molecular function | 11 | 82 | 937 | 17548 | 2.51227321237994 | 0.004120966911596 | 0.0635806323503383 | 2.38500087253413 | 0.0117395944503735 | CTNND2//ESR1//AMER2//CD2AP//AR//NR4A2//SOX17//SKP1//TBL1XR1//TCF7L1//KLF4// |
| GO:0004860 | protein kinase inhibitor activity | Molecular function | 12 | 95 | 937 | 17548 | 2.3656237712745 | 0.00460516233599012 | 0.0694727297508961 | 2.33675505593791 | 0.0128068303094984 | CDKN1A//KAT2B//PRKAR2B//YWHAG//CAMK2N2//SPRED1//FLRT1//NCK1//TAOK3//ASPN//DUS2//SOCS5// |
| GO:0045296 | cadherin binding | Molecular function | 29 | 324 | 937 | 17548 | 1.67625861364744 | 0.00461007311618601 | 0.0694727297508961 | 2.33629218660508 | 0.0309498399146211 | CNN3//TMOD3//CRKL//MB21D2//DDX6//EPHA2//EPS15//DOCK9//CD2AP//TES//SNX5//ARFIP1//GOLGA3//CDH20//IDH1//ITGA6//ARHGAP1//MYO1B//NCK1//LIMA1//PFKP//CC2D1A//PAK6//TWF1//RPL15//SNX2//AHNAK//CALD1//SLK// |
| GO:0050839 | cell adhesion molecule binding | Molecular function | 40 | 489 | 937 | 17548 | 1.53193086756018 | 0.00493273819631251 | 0.0734807896830001 | 2.30691193398324 | 0.0426894343649947 | ADAM10//NPNT//NRG1//IGF1//ITGA6//ITGA5//ITGAV//KDR//ADAM9//CRKL//MB21D2//DDX6//EPHA2//EPS15//DOCK9//CD2AP//TES//SNX5//ARFIP1//GOLGA3//CDH20//IDH1//ARHGAP1//MYO1B//NCK1//LIMA1//PFKP//CC2D1A//PAK6//TWF1//RPL15//SNX2//AHNAK//CALD1//SLK//CNN3//TMOD3//CADM2//NPTN//MSN// |
| GO:0000900 | translation repressor activity, mRNA regulatory element binding | Molecular function | 4 | 14 | 937 | 17548 | 5.3508156731209 | 0.00525581819594246 | 0.0748520921092465 | 2.27935966587304 | 0.00426894343649947 | CPEB2//CPEB3//CPEB1//CPEB4// |
| GO:0005095 | GTPase inhibitor activity | Molecular function | 4 | 14 | 937 | 17548 | 5.3508156731209 | 0.00525581819594246 | 0.0748520921092465 | 2.27935966587304 | 0.00426894343649947 | IQGAP2//CPEB2//IPO5//SLIT2// |
| GO:0033549 | MAP kinase phosphatase activity | Molecular function | 4 | 14 | 937 | 17548 | 5.3508156731209 | 0.00525581819594246 | 0.0748520921092465 | 2.27935966587304 | 0.00426894343649947 | DUSP10//DUSP1//DUSP2//DUSP5// |
| GO:0035259 | glucocorticoid receptor binding | Molecular function | 4 | 14 | 937 | 17548 | 5.3508156731209 | 0.00525581819594246 | 0.0748520921092465 | 2.27935966587304 | 0.00426894343649947 | CEBPB//NR4A2//STAT3//NR4A3// |
| GO:1901981 | phosphatidylinositol phosphate binding | Molecular function | 16 | 147 | 937 | 17548 | 2.03840597071272 | 0.00531921086122563 | 0.074931492132048 | 2.27415279327566 | 0.0170757737459979 | FCHO2//AMER2//MYO1B//PFN2//TWF1//SESTD1//IQGAP2//ARAP2//PLEKHB2//PLEKHF2//PLEK//PLEKHA1//OSBPL5//ARFIP1//PLEKHA3//DENND1B// |
| GO:0004702 | signal transducer, downstream of receptor, with serine/threonine kinase activity | Molecular function | 14 | 123 | 937 | 17548 | 2.13162575595873 | 0.00597086459512274 | 0.0832068872610653 | 2.22396277746297 | 0.0149413020277481 | MAPK1//MAP3K2//EPHA2//EPHA4//EPHA5//EPHA7//KDR//MAP3K5//MET//TAOK3//MINK1//BMPR2//TGFBR2//ACVR1// |
| GO:0140096 | catalytic activity, acting on a protein | Molecular function | 153 | 2370 | 937 | 17548 | 1.20901341474947 | 0.0062808607848762 | 0.0865956976297825 | 2.20198083261569 | 0.163287086446105 | TMX3//ADAM10//NPEPL1//CTSA//MMP2//NLN//THOP1//ADAM12//ADAM18//ADAM9//LMLN//F3//ARID4B//MIER1//ARID4A//RBBP7//REST//BRMS1L//ASPH//HIPK3//CDK8//MAP3K2//EPHA4//MMD//PAN3//MAP3K5//MINK1//CDK17//PRKAA1//CCL2//CCL8//MAP2K4//ACVR1//PKDCC//RPS6KA5//STK17B//FASTK//OSR1//PDIK1L//DSTYK//LATS2//MKNK2//TAOK3//PRKACB//MAPK1//PAK6//FAM20C//STK33//MASTL//SQSTM1//CCNC//RPS6KA4//ULK2//SLK//TGFBR2//HMGA2//CDK6//CDKN1A//CDK19//BMPR2//EPHA2//EPHA5//EPHA7//KDR//MET//EREG//NRG1//JAK1//GRB2//PCMTD1//CTDSPL2//PPP2CA//PTEN//CDC14A//PPM1L//PPM1E//PPP6C//PPM1F//PPP3R1//PTPN21//DUSP10//PTPDC1//DUSP2//DUSP5//PTPRG//PTP4A1//DUSP1//PTPN4//TOPORS//WWP2//KLHL2//HECTD2//RNF38//ZNRF2//FBXL5//KLHL3//MYLIP//RNF141//RLIM//UBE2W//FBXW7//KLHL14//MARCH4//RNF2//RNF4//SKP1//KLHL15//FBXO30//KBTBD8//CDC23//LONRF1//OTUD3//USP27X//ATXN3//USP28//USP46//USP45//JOSD1//USP3//IMMP2L//ZFYVE9//PTAR1//LYPLA1//PPT2//EZH1//PRDM16//DPY30//KMT2B//ZDHHC5//ZDHHC1//ATG16L1//PIAS4//UBE2I//CCDC126//XYLT2//EGLN1//EGLN3//KDM2A//ARID5B//USP24//WDR20//TRIM3//RNF145//RNF180//MDM4//ANKIB1//HECW2//AKTIP//UBE2Z//RNF128//LONRF3//UBE3B//SOSTDC1// |
| GO:0046983 | protein dimerization activity | Molecular function | 89 | 1286 | 937 | 17548 | 1.2960957093135 | 0.00665402892292141 | 0.0905616366128939 | 2.17691531572481 | 0.0949839914621131 | ADAM10//CALCOCO2//CEBPB//ACOT7//DYNLL2//SYT6//GADD45A//FMR1//CADM2//CDH20//GRHL1//HPRT1//IDH1//INHBB//JUN//RBM44//FIBIN//LRP6//MEF2D//MAP3K5//NEUROG1//NR4A2//CHMP3//PKD2//PLEK//UGT1A10//UGT1A8//UGT1A7//UGT1A6//UGT1A9//UGT1A4//UGT1A1//UGT1A3//DSCAML1//ZBTB4//CLIP1//DMRTA1//BNIP3//SNX2//STAT3//TFAP2A//TGFB2//TSG101//VEGFB//BHLHE41//NR4A3//UXS1//FXR1//SBF2//GNPTG//DPY30//BHLHE40//SQSTM1//ACVR1//NOG//SLIT2//HAND1//HAND2//CD69//SLK//BCL2L11//HMG20A//ADRB1//DR1//SNX5//H2AFZ//NEUROD1//P2RY1//PPARG//PPP2CA//RRAGD//SOX17//SOS1//SOS2//SOX4//TEAD1//TTR//ADIPOR2//E2F1//E2F3//E2F5//STX2//SLC24A2//AR//NHLH2//NPAS2//TCF21//TFAP2C//CLOCK// |
| GO:0045182 | translation regulator activity | Molecular function | 8 | 53 | 937 | 17548 | 2.8268460159884 | 0.00670826937873288 | 0.0905616366128939 | 2.17338950609155 | 0.00853788687299893 | CPEB2//CPEB3//CPEB1//CPEB4//FMR1//NANOS1//PAIP2//FXR1// |
| GO:0034483 | heparan sulfate sulfotransferase activity | Molecular function | 4 | 15 | 937 | 17548 | 4.99409462824618 | 0.0068678572627716 | 0.0917602372428041 | 2.16317873952085 | 0.00426894343649947 | HS2ST1//HS3ST5//HS3ST3A1//HS3ST1// |
| GO:0001190 | transcriptional activator activity, RNA polymerase II transcription factor binding | Molecular function | 9 | 65 | 937 | 17548 | 2.59308759543551 | 0.00737249795351203 | 0.0966711803125761 | 2.13238533932938 | 0.0096051227321238 | ZFPM2//NEUROD1//POU3F2//SOX4//SOX11//WWP2//JUN//KLF4//CLOCK// |
| GO:0004721 | phosphoprotein phosphatase activity | Molecular function | 18 | 179 | 937 | 17548 | 1.88324797433864 | 0.00738460405165512 | 0.0966711803125761 | 2.13167278576723 | 0.0192102454642476 | PPM1L//PPM1E//PPP2CA//PPP6C//PTEN//CDC14A//PPM1F//PPP3R1//PTPN21//DUSP10//PTPDC1//DUSP2//DUSP5//PTPRG//PTP4A1//DUSP1//PTPN4//CTDSPL2// |
| GO:0035258 | steroid hormone receptor binding | Molecular function | 11 | 89 | 937 | 17548 | 2.31467869005792 | 0.00767734511181091 | 0.0994983926490694 | 2.11478893656049 | 0.0117395944503735 | ESR1//PPARG//STRN//CEBPB//NR4A2//STAT3//NR4A3//FHL2//RNF4//TCF21//TGFB1I1// |
| GO:0042803 | protein homodimerization activity | Molecular function | 60 | 819 | 937 | 17548 | 1.37200401874895 | 0.00792554836201705 | 0.101698125516575 | 2.10097067960117 | 0.064034151547492 | ADAM10//CALCOCO2//CEBPB//ACOT7//DYNLL2//SYT6//GADD45A//FMR1//CADM2//CDH20//GRHL1//HPRT1//IDH1//INHBB//JUN//RBM44//FIBIN//LRP6//MEF2D//MAP3K5//NEUROG1//NR4A2//CHMP3//PKD2//PLEK//UGT1A10//UGT1A8//UGT1A7//UGT1A6//UGT1A9//UGT1A4//UGT1A1//UGT1A3//DSCAML1//ZBTB4//CLIP1//DMRTA1//BNIP3//SNX2//STAT3//TFAP2A//TGFB2//TSG101//VEGFB//BHLHE41//NR4A3//UXS1//FXR1//SBF2//GNPTG//DPY30//BHLHE40//SQSTM1//ACVR1//NOG//SLIT2//HAND1//HAND2//CD69//SLK// |
| GO:0005088 | Ras guanyl-nucleotide exchange factor activity | Molecular function | 23 | 251 | 937 | 17548 | 1.71609825373001 | 0.00828406923732706 | 0.105256409133097 | 2.08175627997856 | 0.0245464247598719 | FGD4//FGD5//ARHGEF4//ARHGEF28//SOS1//SOS2//RGL1//DENND5B//DENND1B//TRAPPC8//RAB3GAP1//SBF2//EPS8//RANBP9//RAPGEF4//ADRB1//EREG//RASGEF1A//GRB2//NRG1//IL2//JAK1//MET// |
| GO:0019902 | phosphatase binding | Molecular function | 17 | 168 | 937 | 17548 | 1.89508055089699 | 0.00853411828166977 | 0.107380750417903 | 2.06884134217503 | 0.0181430096051227 | AKAP11//GRB2//JAK1//MAP3K5//MET//PPARG//MTMR9//STAT3//STRN//MASTL//CRY2//SPRED1//PPP1R12A//PPP1R12C//PPP3R1//MAPK1//SBF2// |
| GO:0005057 | signal transducer activity, downstream of receptor | Molecular function | 17 | 169 | 937 | 17548 | 1.88386705651298 | 0.00903240182624057 | 0.112557622757767 | 2.04419675010909 | 0.0181430096051227 | BMPR2//TGFBR2//ACVR1//MAPK1//MAP3K2//EPHA2//EPHA4//EPHA5//EPHA7//KDR//MAP3K5//MET//TAOK3//MINK1//ADRB1//FLRT1//NSMAF// |
| GO:0016757 | transferase activity, transferring glycosyl groups | Molecular function | 25 | 283 | 937 | 17548 | 1.65440413921583 | 0.00952073973362074 | 0.117513130426405 | 2.02132930689403 | 0.0266808964781217 | ST6GALNAC3//ST6GALNAC6//ST8SIA5//ST8SIA3//WDFY3//ST6GAL2//ST3GAL3//ST3GAL5//HPRT1//PIGA//UGT1A10//UGT1A8//UGT1A7//UGT1A6//UGT1A5//UGT1A9//UGT1A4//UGT1A1//UGT1A3//XYLT2//B3GNT5//B3GALT2//FUT9//CCDC126//KDELC1// |
| GO:0016772 | transferase activity, transferring phosphorus-containing groups | Molecular function | 71 | 1012 | 937 | 17548 | 1.31391076558354 | 0.0108813409355555 | 0.13198566832139 | 1.96331758211689 | 0.0757737459978655 | PIKFYVE//ZFYVE21//PTGES3//PFKP//REV3L//POLR3G//DGKH//SPHK2//DGKE//GNPTG//UGP2//CMPK1//CDS1//MPP1//NME1//HIPK3//CDK8//MAP3K2//EPHA4//MMD//PAN3//MAP3K5//MINK1//CDK17//PRKAA1//CCL2//CCL8//MAP2K4//ACVR1//PKDCC//RPS6KA5//STK17B//FASTK//OSR1//PDIK1L//DSTYK//LATS2//MKNK2//TAOK3//PRKACB//MAPK1//PAK6//FAM20C//STK33//MASTL//SQSTM1//CCNC//RPS6KA4//ULK2//SLK//TGFBR2//HMGA2//CDK6//CDKN1A//CDK19//BMPR2//EPHA2//EPHA5//EPHA7//KDR//MET//EREG//NRG1//JAK1//GRB2//PIK3CB//GAB1//PIK3R3//NAGK//ESR1//SOSTDC1// |
| GO:0004675 | transmembrane receptor protein serine/threonine kinase activity | Molecular function | 4 | 17 | 937 | 17548 | 4.40655408374663 | 0.0109988056934492 | 0.13198566832139 | 1.9586544701967 | 0.00426894343649947 | TGFBR2//ACVR1//BMPR2//SOSTDC1// |
| GO:0005005 | transmembrane-ephrin receptor activity | Molecular function | 4 | 17 | 937 | 17548 | 4.40655408374663 | 0.0109988056934492 | 0.13198566832139 | 1.9586544701967 | 0.00426894343649947 | EPHA2//EPHA4//EPHA5//EPHA7// |
| GO:0001158 | enhancer sequence-specific DNA binding | Molecular function | 11 | 94 | 937 | 17548 | 2.19155748313994 | 0.0114237159682669 | 0.135826934815357 | 1.94219260344499 | 0.0117395944503735 | ARX//FLI1//NR5A2//H2AFZ//JUN//MEOX2//PBX3//POU4F1//SMARCD2//SLC2A4RG//SOX11// |
| GO:0019787 | ubiquitin-like protein transferase activity | Molecular function | 37 | 472 | 937 | 17548 | 1.46807336794313 | 0.0126842316105188 | 0.146817489762868 | 1.89673583647687 | 0.0394877267876201 | TOPORS//WWP2//KLHL2//HECTD2//RNF38//ZNRF2//FBXL5//KLHL3//MYLIP//RNF141//RLIM//UBE2W//FBXW7//KLHL14//MARCH4//RNF2//RNF4//SKP1//KLHL15//FBXO30//KBTBD8//CDC23//LONRF1//PIAS4//UBE2I//TRIM3//RNF145//RNF180//MDM4//ANKIB1//HECW2//AKTIP//UBE2Z//RNF128//LONRF3//UBE3B//ATG16L1// |
| GO:0048306 | calcium-dependent protein binding | Molecular function | 8 | 59 | 937 | 17548 | 2.53937014995568 | 0.0126908235976225 | 0.146817489762868 | 1.89651019253917 | 0.00853788687299893 | STMN2//SYT6//STX2//NSMF//SNAP25//TSG101//VLDLR//WFS1// |
| GO:0019199 | transmembrane receptor protein kinase activity | Molecular function | 10 | 83 | 937 | 17548 | 2.2563680549305 | 0.0127692820594529 | 0.146817489762868 | 1.89383351983784 | 0.0106723585912487 | TGFBR2//ACVR1//EPHA2//EPHA4//EPHA5//EPHA7//KDR//MET//BMPR2//SOSTDC1// |
| GO:0008194 | UDP-glycosyltransferase activity | Molecular function | 15 | 148 | 937 | 17548 | 1.89809339755978 | 0.0128012163142007 | 0.146817489762868 | 1.8927487636764 | 0.016008537886873 | WDFY3//XYLT2//B3GNT5//B3GALT2//UGT1A10//UGT1A8//UGT1A7//UGT1A6//UGT1A5//UGT1A9//UGT1A4//UGT1A1//UGT1A3//PIGA//CCDC126// |
| GO:0008301 | DNA binding, bending | Molecular function | 4 | 18 | 937 | 17548 | 4.16174552353848 | 0.0135546505042845 | 0.154094974153971 | 1.86791167587158 | 0.00426894343649947 | FOXC1//HMGB3//TRERF1//HMGA2// |
| GO:0016723 | oxidoreductase activity, oxidizing metal ions, NAD or NADP as acceptor | Molecular function | 3 | 10 | 937 | 17548 | 5.61835645677695 | 0.0137272429340591 | 0.154700059500353 | 1.8624166804443 | 0.0032017075773746 | CYBRD1//STEAP2//MTRR// |
| GO:0031267 | small GTPase binding | Molecular function | 43 | 570 | 937 | 17548 | 1.41280308562227 | 0.0142202635016065 | 0.158874668086914 | 1.84709235605116 | 0.0458911419423693 | RANBP9//RAPGEF4//ADRB1//EREG//RASGEF1A//GRB2//NRG1//IL2//JAK1//MET//SOS1//FGD4//FGD5//ARHGEF4//ARHGEF28//SOS2//RGL1//IPO5//SPHK2//IQGAP2//CDC42EP3//DENND5B//DENND1B//TRAPPC8//RAB3GAP1//SBF2//TBC1D20//TBC1D12//ARHGAP1//NSF//EVI5//TBC1D17//USP6NL//EXOC5//EPS8//PAK6//ARAP2//APPL1//AGFG1//AGFG2//PEX5L//ERRFI1//GIT2// |
| GO:0005160 | transforming growth factor beta receptor binding | Molecular function | 7 | 49 | 937 | 17548 | 2.67540783656045 | 0.0146472782950502 | 0.162246774960556 | 1.83424306686303 | 0.00747065101387407 | BAMBI//TGFB2//TGFBR2//GDF11//INHBB//BMP3//RASL11B// |
| GO:0005003 | ephrin receptor activity | Molecular function | 4 | 19 | 937 | 17548 | 3.94270628545751 | 0.0164584284615305 | 0.180763756662233 | 1.78361163589588 | 0.00426894343649947 | EPHA4//EPHA5//EPHA7//EPHA2// |
| GO:0004725 | protein tyrosine phosphatase activity | Molecular function | 11 | 100 | 937 | 17548 | 2.06006403415155 | 0.017598806632128 | 0.191093120069586 | 1.75451678051842 | 0.0117395944503735 | DUSP1//PTPN4//PTPRG//PTPN21//DUSP10//PTPDC1//DUSP2//DUSP5//PTEN//PTP4A1//CDC14A// |
| GO:0000980 | RNA polymerase II distal enhancer sequence-specific DNA binding | Molecular function | 9 | 75 | 937 | 17548 | 2.24734258271078 | 0.0181026941608824 | 0.191093120069586 | 1.74225678578649 | 0.0096051227321238 | ARX//FLI1//NR5A2//H2AFZ//JUN//MEOX2//PBX3//POU4F1//SMARCD2// |
| GO:0005172 | vascular endothelial growth factor receptor binding | Molecular function | 3 | 11 | 937 | 17548 | 5.10759677888813 | 0.0181361525991968 | 0.191093120069586 | 1.74145483867387 | 0.0032017075773746 | VEGFB//ITGA5//CD2AP// |
| GO:0015924 | mannosyl-oligosaccharide mannosidase activity | Molecular function | 3 | 11 | 937 | 17548 | 5.10759677888813 | 0.0181361525991968 | 0.191093120069586 | 1.74145483867387 | 0.0032017075773746 | MAN1A1//EDEM1//MAN2A1// |
| GO:0016004 | phospholipase activator activity | Molecular function | 3 | 11 | 937 | 17548 | 5.10759677888813 | 0.0181361525991968 | 0.191093120069586 | 1.74145483867387 | 0.0032017075773746 | PLAA//NSMAF//CCL8// |
| GO:0001010 | transcription factor activity, sequence-specific DNA binding transcription factor recruiting | Molecular function | 4 | 20 | 937 | 17548 | 3.74557097118463 | 0.0197231286747151 | 0.206138506148635 | 1.70502419191061 | 0.00426894343649947 | PPARG//RARB//THRA//KLF4// |
| GO:0008146 | sulfotransferase activity | Molecular function | 7 | 52 | 937 | 17548 | 2.52105738445119 | 0.0198915073548923 | 0.206235148255523 | 1.70133230530655 | 0.00747065101387407 | CHST7//CHST1//HS2ST1//HS3ST5//HS3ST3A1//HS3ST1//DSEL// |
| GO:0008047 | enzyme activator activity | Molecular function | 39 | 520 | 937 | 17548 | 1.40458911419424 | 0.0205158913917747 | 0.209576673743131 | 1.68790960869372 | 0.0416221985058698 | DNAJB4//FAM13A//DLC1//CDC42EP3//GNB5//ARAP2//TAGAP//TBC1D20//RAB3GAP1//TBC1D12//APPL1//RGS17//GNAQ//AGFG1//AGFG2//JUN//ARHGAP1//ARHGAP6//ERRFI1//ELMOD1//RGS3//SOS1//EVI5//TBC1D17//TRIP10//ARHGAP12//USP6NL//GIT2//CCL8//PLAA//NSMAF//PCOLCE2//IL2//LAMTOR3//CDKN1A//NRG1//EFNA5//CALM2//CTSA// |
| GO:0005543 | phospholipid binding | Molecular function | 32 | 410 | 937 | 17548 | 1.46168623265742 | 0.0205372203436556 | 0.209576673743131 | 1.68745833732604 | 0.0341515474919957 | OSBPL5//FCHO2//SYT6//WDFY3//ZFYVE9//AMER2//MYO1B//PFN2//TWF1//SESTD1//IQGAP2//ARAP2//PLEKHB2//CHMP3//PLEKHF2//SNX5//PXK//RUFY2//PLEKHA1//AIDA//SNX2//SBF2//LPAR1//PLEK//ARFIP1//PLEKHA3//DENND1B//NUP35//F3//LPAR3//NR5A2//PSD// |
| GO:0001221 | transcription cofactor binding | Molecular function | 6 | 41 | 937 | 17548 | 2.74066168623266 | 0.0207275994915407 | 0.20986694485185 | 1.68345099158409 | 0.0064034151547492 | PER2//ESR1//FOXC1//TEAD1//NR4A3//KLF4// |
| GO:0019887 | protein kinase regulator activity | Molecular function | 18 | 200 | 937 | 17548 | 1.68550693703308 | 0.0212559333511382 | 0.213547981574226 | 1.67251982034403 | 0.0192102454642476 | CDKN1A//FLRT1//NCK1//TAOK3//ASPN//DUS2//SOCS5//KAT2B//PRKAR2B//YWHAG//CAMK2N2//CCNY//CCNC//CCNE2//SPRED1//NRG1//EFNA5//CALM2// |
| GO:0017147 | Wnt-protein binding | Molecular function | 5 | 31 | 937 | 17548 | 3.02062175095535 | 0.0229661042580427 | 0.224251284368659 | 1.63891266796237 | 0.00533617929562433 | FZD10//APCDD1//FRZB//LRP6//FZD6// |
| GO:0019208 | phosphatase regulator activity | Molecular function | 10 | 91 | 937 | 17548 | 2.05800602812342 | 0.0231157548971614 | 0.224251284368659 | 1.63609191912755 | 0.0106723585912487 | PPP1R37//URI1//ANP32E//UBXN2B//PPP4R2//PPP1R15B//CALM2//PPP1R12A//PPP1R12C//SBF2// |
| GO:0005165 | neurotrophin receptor binding | Molecular function | 3 | 12 | 937 | 17548 | 4.68196371398079 | 0.0232378258796458 | 0.224251284368659 | 1.63380450677402 | 0.0032017075773746 | NTF3//EFNA5//GRB2// |
| GO:0035925 | mRNA 3'-UTR AU-rich region binding | Molecular function | 3 | 12 | 937 | 17548 | 4.68196371398079 | 0.0232378258796458 | 0.224251284368659 | 1.63380450677402 | 0.0032017075773746 | CPEB2//CPEB3//CPEB1// |
| GO:0001223 | transcription coactivator binding | Molecular function | 4 | 21 | 937 | 17548 | 3.56721044874727 | 0.023359508788402 | 0.224251284368659 | 1.63153629395388 | 0.00426894343649947 | ESR1//FOXC1//TEAD1//NR4A3// |
| GO:0045499 | chemorepellent activity | Molecular function | 4 | 21 | 937 | 17548 | 3.56721044874727 | 0.023359508788402 | 0.224251284368659 | 1.63153629395388 | 0.00426894343649947 | SEMA4B//EFNA5//EPHA7//NRG1// |
| GO:0004842 | ubiquitin-protein transferase activity | Molecular function | 34 | 446 | 937 | 17548 | 1.42768400246948 | 0.0236259208714456 | 0.224872190843108 | 1.62661125492057 | 0.0362860192102455 | TOPORS//TRIM3//WWP2//HECTD2//RNF38//RNF145//ZNRF2//RNF180//MYLIP//MDM4//RLIM//ANKIB1//UBE2W//HECW2//RNF2//RNF4//AKTIP//SKP1//UBE2Z//RNF128//LONRF3//FBXO30//UBE3B//LONRF1//KLHL2//FBXL5//KLHL3//RNF141//FBXW7//KLHL14//MARCH4//KLHL15//KBTBD8//CDC23// |
| GO:0003730 | mRNA 3'-UTR binding | Molecular function | 8 | 66 | 937 | 17548 | 2.27004301283917 | 0.0237712115320261 | 0.224872190843108 | 1.62394868330063 | 0.00853788687299893 | CPEB2//CPEB3//CPEB1//ELAVL2//FMR1//CPEB4//FXR1//SECISBP2L// |
| GO:0005326 | neurotransmitter transporter activity | Molecular function | 7 | 54 | 937 | 17548 | 2.42768488873078 | 0.0240396440704736 | 0.22576361387923 | 1.61907196675971 | 0.00747065101387407 | SLC17A7//SLC17A6//SLC6A14//SLC6A6//SLC6A1//SLC32A1//SLC44A1// |
| GO:0005126 | cytokine receptor binding | Molecular function | 23 | 278 | 937 | 17548 | 1.54942684059796 | 0.0250934158823827 | 0.233964510673151 | 1.60044021561272 | 0.0245464247598719 | BAMBI//TGFB2//JAK1//IFNA1//IL2//OSM//GDF11//INHBB//BMP3//RASL11B//TNF//NTF3//EFNA5//GRB2//CD2AP//SPRED1//CCL2//CCL8//CXCL14//STAT3//TGFBR2//VEGFB//ITGA5// |
| GO:0004407 | histone deacetylase activity | Molecular function | 6 | 43 | 937 | 17548 | 2.6131890496637 | 0.0257013451435955 | 0.237921023614998 | 1.59004414619582 | 0.0064034151547492 | ARID4B//MIER1//ARID4A//RBBP7//REST//BRMS1L// |
| GO:0042813 | Wnt-activated receptor activity | Molecular function | 4 | 22 | 937 | 17548 | 3.40506451925876 | 0.0273761178415358 | 0.25162729590518 | 1.56262813836191 | 0.00426894343649947 | FZD10//FRZB//LRP6//FZD6// |
| GO:0004709 | MAP kinase kinase kinase activity | Molecular function | 9 | 81 | 937 | 17548 | 2.08087276176924 | 0.0284145977378265 | 0.254067600453714 | 1.54645848783248 | 0.0096051227321238 | MAP3K2//EPHA2//EPHA4//EPHA5//EPHA7//KDR//MAP3K5//MET//TAOK3// |
| GO:0033558 | protein deacetylase activity | Molecular function | 6 | 44 | 937 | 17548 | 2.55379838944407 | 0.0284617868030553 | 0.254067600453714 | 1.54573783881433 | 0.0064034151547492 | ARID4B//MIER1//ARID4A//RBBP7//REST//BRMS1L// |
| GO:0045309 | protein phosphorylated amino acid binding | Molecular function | 6 | 44 | 937 | 17548 | 2.55379838944407 | 0.0284617868030553 | 0.254067600453714 | 1.54573783881433 | 0.0064034151547492 | CRK//CRKL//GRB2//MAPK1//PIK3R3//FBXW7// |
| GO:0060229 | lipase activator activity | Molecular function | 3 | 13 | 937 | 17548 | 4.32181265905919 | 0.0290337642785062 | 0.254067600453714 | 1.53709665347589 | 0.0032017075773746 | CCL8//PLAA//NSMAF// |
| GO:0035326 | enhancer binding | Molecular function | 11 | 108 | 937 | 17548 | 1.90746669828847 | 0.0293138605218735 | 0.254067600453714 | 1.53292698289418 | 0.0117395944503735 | ARX//FLI1//NR5A2//H2AFZ//JUN//MEOX2//PBX3//POU4F1//SMARCD2//SLC2A4RG//SOX11// |
| GO:0005154 | epidermal growth factor receptor binding | Molecular function | 5 | 33 | 937 | 17548 | 2.83755376604896 | 0.0294059722747354 | 0.254067600453714 | 1.53156445657578 | 0.00533617929562433 | EREG//GRB2//ITGA5//SNX2//SOCS5// |
| GO:0016538 | cyclin-dependent protein serine/threonine kinase regulator activity | Molecular function | 5 | 33 | 937 | 17548 | 2.83755376604896 | 0.0294059722747354 | 0.254067600453714 | 1.53156445657578 | 0.00533617929562433 | CDKN1A//KAT2B//CCNY//CCNC//CCNE2// |
| GO:0016896 | exoribonuclease activity, producing 5'-phosphomonoesters | Molecular function | 5 | 33 | 937 | 17548 | 2.83755376604896 | 0.0294059722747354 | 0.254067600453714 | 1.53156445657578 | 0.00533617929562433 | CNOT7//DCP2//CNOT6L//PAN3//CNOT6// |
| GO:0030291 | protein serine/threonine kinase inhibitor activity | Molecular function | 5 | 33 | 937 | 17548 | 2.83755376604896 | 0.0294059722747354 | 0.254067600453714 | 1.53156445657578 | 0.00533617929562433 | CDKN1A//KAT2B//PRKAR2B//YWHAG//SPRED1// |
| GO:0051427 | hormone receptor binding | Molecular function | 16 | 179 | 937 | 17548 | 1.67399819941213 | 0.0303299940074435 | 0.259761872419242 | 1.51812767549661 | 0.0170757737459979 | JAK1//ESR1//PPARG//STRN//TACC2//CEBPB//NR4A2//STAT3//NR4A3//TOB2//RARB//FHL2//RNF4//TCF21//TGFB1I1//PTHLH// |
| GO:0035591 | signaling adaptor activity | Molecular function | 9 | 82 | 937 | 17548 | 2.05549626467449 | 0.0304658986170716 | 0.259761872419242 | 1.51618600747288 | 0.0096051227321238 | TOB1//CRK//CRKL//EPS8//GAB1//GRB2//ARHGAP1//ARHGAP6//NCK1// |
| GO:0035257 | nuclear hormone receptor binding | Molecular function | 14 | 151 | 937 | 17548 | 1.73635740386043 | 0.0313900165792965 | 0.265891905142276 | 1.50320845491815 | 0.0149413020277481 | ESR1//PPARG//STRN//CEBPB//NR4A2//STAT3//NR4A3//TOB2//RARB//FHL2//RNF4//TCF21//TGFB1I1//TACC2// |
| GO:0051087 | chaperone binding | Molecular function | 10 | 96 | 937 | 17548 | 1.95081821415866 | 0.0320675683798102 | 0.269867328702818 | 1.49393397051335 | 0.0106723585912487 | GNB5//PTGES3//DNAJB4//DNAJB5//SACS//DNAJB9//CDC37L1//TSC1//BAG5//FNIP1// |
| GO:0016791 | phosphatase activity | Molecular function | 22 | 270 | 937 | 17548 | 1.52597335863078 | 0.0324467484541961 | 0.271296683849278 | 1.48882881809794 | 0.0234791889007471 | PTEN//MTMR9//CTDSPL2//PPP2CA//CDC14A//PPM1L//PPM1E//PPP6C//PPM1F//PPP3R1//PTPN21//DUSP10//PTPDC1//DUSP2//DUSP5//PTPRG//PTP4A1//DUSP1//PTPN4//SGPP1//PIKFYVE//HDHD2// |
| GO:0005501 | retinoid binding | Molecular function | 5 | 34 | 937 | 17548 | 2.75409630234164 | 0.0330117715551521 | 0.274251640612033 | 1.48133116891469 | 0.00533617929562433 | UGT1A8//UGT1A7//UGT1A9//UGT1A1//UGT1A3// |
| GO:0004722 | protein serine/threonine phosphatase activity | Molecular function | 8 | 71 | 937 | 17548 | 2.11018082883641 | 0.0349803159641501 | 0.287701135183745 | 1.45617627201952 | 0.00853788687299893 | PPP3R1//PPM1F//PPM1L//PPM1E//PPP2CA//PPP6C//PTEN//CDC14A// |
| GO:0004857 | enzyme inhibitor activity | Molecular function | 30 | 397 | 937 | 17548 | 1.41520313772719 | 0.0353514902493949 | 0.287701135183745 | 1.45159227370733 | 0.032017075773746 | CDKN1A//FLRT1//NCK1//TAOK3//ASPN//DUS2//SOCS5//KAT2B//PRKAR2B//PPP1R37//URI1//RECK//SERPINI1//SPOCK1//IQGAP2//CPEB2//IPO5//SLIT2//YWHAG//CAMK2N2//LRP6//ANP32E//SPRED1//FNIP1//FRY//PPP1R12A//UGT1A8//UGT1A7//UGT1A1//PPP1R12C// |
| GO:0005523 | tropomyosin binding | Molecular function | 3 | 14 | 937 | 17548 | 4.01311175484068 | 0.0355186586646599 | 0.287701135183745 | 1.44954344338713 | 0.0032017075773746 | TMOD3//TMOD1//CALD1// |
| GO:0030546 | receptor activator activity | Molecular function | 3 | 14 | 937 | 17548 | 4.01311175484068 | 0.0355186586646599 | 0.287701135183745 | 1.44954344338713 | 0.0032017075773746 | EFNA5//NRG1//IGF1// |
| GO:0030552 | cAMP binding | Molecular function | 4 | 24 | 937 | 17548 | 3.12130914265386 | 0.0365734659522084 | 0.288485392151948 | 1.43683388099111 | 0.00426894343649947 | PDE10A//RAPGEF4//PRKAR2B//HCN2// |
| GO:0001784 | phosphotyrosine residue binding | Molecular function | 5 | 35 | 937 | 17548 | 2.67540783656045 | 0.0368808788687443 | 0.288485392151948 | 1.4331987382882 | 0.00533617929562433 | CRK//CRKL//GRB2//MAPK1//PIK3R3// |
| GO:0004532 | exoribonuclease activity | Molecular function | 5 | 35 | 937 | 17548 | 2.67540783656045 | 0.0368808788687443 | 0.288485392151948 | 1.4331987382882 | 0.00533617929562433 | CNOT7//DCP2//CNOT6L//PAN3//CNOT6// |
| GO:0004693 | cyclin-dependent protein serine/threonine kinase activity | Molecular function | 5 | 35 | 937 | 17548 | 2.67540783656045 | 0.0368808788687443 | 0.288485392151948 | 1.4331987382882 | 0.00533617929562433 | CDK6//CDK8//CDKN1A//CDK19//CDK17// |
| GO:0019840 | isoprenoid binding | Molecular function | 5 | 35 | 937 | 17548 | 2.67540783656045 | 0.0368808788687443 | 0.288485392151948 | 1.4331987382882 | 0.00533617929562433 | UGT1A8//UGT1A7//UGT1A9//UGT1A1//UGT1A3// |
| GO:0008289 | lipid binding | Molecular function | 49 | 707 | 937 | 17548 | 1.29797013852933 | 0.0369510610318082 | 0.288485392151948 | 1.43237308653455 | 0.0522945570971185 | OSBPL5//FCHO2//SYT6//UGT1A8//UGT1A7//UGT1A9//UGT1A1//UGT1A3//PGRMC2//ESR1//AR//NR3C2//NUP35//F3//LPAR3//NR5A2//ARFIP1//PSD//WDFY3//ZFYVE9//AMER2//MYO1B//PFN2//TWF1//SESTD1//IQGAP2//ARAP2//PLEKHB2//CHMP3//PLEKHF2//SNX5//PXK//RUFY2//PLEKHA1//AIDA//SNX2//SBF2//LPAR1//IL2//PLEK//S1PR1//PPARG//PLEKHA3//DENND1B//DLC1//EPN1//PTEN//ACBD5//TRIP10// |
| GO:0097159 | organic cyclic compound binding | Molecular function | 358 | 6221 | 937 | 17548 | 1.07773220357185 | 0.0382658297911109 | 0.296961170115447 | 1.41718886459526 | 0.382070437566702 | HPRT1//REV3L//HMGB3//BAG5//CPEB2//CPEB3//CPEB1//CPEB4//CRY2//BAHD1//NR5A2//ZNF568//MEF2D//NFE2//PPARG//RARB//SOX4//SOX11//TFAP2A//THRA//PER2//CEBPB//ZMYND11//CREBRF//E2F3//FOXF1//FOXF2//FOXC1//GATA6//PURG//JARID2//NHLH2//NME1//NR4A2//PAX9//KLF3//ZBTB4//TEF//TFAP2C//NR4A3//ARID5B//RUNX3//KAT2B//HAND2//KLF6//EGR2//CC2D1B//ELK3//EN1//ERG//ESR1//ETS1//FLI1//GRHL1//H2AFZ//IRF1//AR//JUN//MEOX2//MYB//MYBL1//NFIA//NFIB//OTX2//ZBTB7A//POU3F2//CC2D1A//RFX7//BCL11B//SMARCD2//STAT3//TGIF1//KLF10//ZNF217//BHLHE41//FOSL1//KLF7//FUBP3//HAND1//CLOCK//USP3//GADD45A//ARX//PBX3//POU4F1//TEAD1//KLF4//RBL2//REST//IRF9//E2F1//NPAS2//HMGA2//ST18//MBNL1//SLC2A4RG//FMR1//FXR1//OSR1//DZIP1//IMPDH1//REXO1//ZNF804A//TSHZ1//TOPORS//HMG20A//KLF2//ZBTB18//ZNF268//KLF12//ZNF597//RBFOX3//ZNF782//ZNF800//GLIS3//DNMT3B//DR1//E2F5//ZNF367//ZBTB9//KDM2A//MYT1L//CIC//ZFPM2//HBP1//ZDHHC1//HIVEP2//AGFG1//ZBTB41//LBR//ZBTB34//NEUROG1//SYCP3//RNF141//PIAS4//ZFR//ZFAND6//BNC2//ZNF532//BDP1//MAPK1//PHTF2//ZNF630//ZNF287//ZNF512B//TSHZ3//ZBTB26//MIER1//ARID4A//RNF4//ATXN1//SOS1//SOS2//STAT4//TMF1//TSG101//ZNF2//ZNF3//ZNF131//ZNF655//DEK//TCF7L1//LCOR//GLYR1//ZIC5//CBFB//BTAF1//ZBTB47//KMT2B//RBBP8//NUP35//NABP1//KHDRBS3//TCERG1//FASTK//CKAP4//CRKL//PABPC5//RBM33//DDX6//ZFC3H1//ELAVL2//ZCCHC24//MORC3//PAN3//FAM133B//TES//GRB2//CNOT7//NANOS1//RBM44//IPO5//ACO1//NOVA1//YTHDF2//RBM47//ZNFX1//CNOT6//RBM25//RBBP7//RPL15//SRSF2//ARHGEF28//ZMAT3//FNDC3B//CAPRIN2//BTF3//UBE2I//YWHAG//AHNAK//SNIP1//ZCCHC3//UNK//RTCA//CCT6A//QKI//SECISBP2L//G3BP2//MSN//DUS2//PAIP2//ESRP1//PGRMC2//NR3C2//UGT1A8//UGT1A1//HIPK3//CDK6//CDK8//MAP3K2//ADCY3//CLCN5//CLCN6//PDIK1L//DGKH//DYNC1LI2//EPHA2//PIKFYVE//EPHA4//EPHA5//EPHA7//ACSL1//CDK19//DSTYK//LATS2//MKNK2//ATAD2//JAK1//KCNJ10//KDR//KIF5A//MAP3K5//MET//MYO1B//NSF//P2RY1//MINK1//CDK17//TAOK3//CMPK1//PFKP//PIK3CB//UBE2W//NAGK//PRKAA1//PRKACB//SPHK2//PAK6//FAM20C//RIMKLB//TWF1//KIF13A//MAP2K4//UBE2Z//BMPR2//STK33//HSPA13//ACTC1//TGFBR2//FYCO1//UBA5//MASTL//DGKE//RPS6KA4//ACVR1//PKDCC//RPS6KA5//STK17B//ITM2B//KIF23//ULK2//SLK//RAB12//RAB21//RAB38//ARL5A//RAB30//GLUD2//GNAQ//NKIRAS1//RHOC//RND3//SAR1B//RAB9B//DNAJC27//RASD1//RAB23//ARL15//RAB22A//RAP2C//RRAGD//RAP1B//RHEB//RASL11B//RAB28//OSBPL5//TRERF1//MTRR//MIEF1//PDE10A//RAPGEF4//PRKAR2B//HCN2//DCTPP1//UGP2//DRD1//MTPN//NKX2-3//EMX2//FOXJ3//LHX6//GTF2E1//MNX1//HOXA3//HOXD1//FOXN2//LHX1//MAF//LHX8//NEUROD1//DMRTA1//PRDM16//SOX17//NKX2-4//SHOX//SNAPC1//ARID4B//TNF//TBL1XR1//HS3ST5//ACOX1//IDH1//ADRB1//DCP2//UXS1//ZEB1//TCF21//BHLHE40// |
| GO:0030674 | protein binding, bridging | Molecular function | 15 | 170 | 937 | 17548 | 1.65245778140498 | 0.0387815111804674 | 0.299171657677891 | 1.41137527198369 | 0.016008537886873 | TOB1//CRK//CRKL//EPS8//GAB1//GRB2//ARHGAP1//ARHGAP6//NCK1//ARRDC4//COL1A2//PPP4R2//FLRT1//NEFH//FBXW7// |
| GO:1901363 | heterocyclic compound binding | Molecular function | 353 | 6135 | 937 | 17548 | 1.0775766526717 | 0.0402223054346205 | 0.308450342267859 | 1.39553304043148 | 0.376734258271078 | HPRT1//REV3L//HMGB3//BAG5//CPEB2//CPEB3//CPEB1//CPEB4//CRY2//BAHD1//NR5A2//ZNF568//MEF2D//NFE2//PPARG//RARB//SOX4//SOX11//TFAP2A//THRA//PER2//CEBPB//ZMYND11//CREBRF//E2F3//FOXF1//FOXF2//FOXC1//GATA6//PURG//JARID2//NHLH2//NME1//NR4A2//PAX9//KLF3//ZBTB4//TEF//TFAP2C//NR4A3//ARID5B//RUNX3//KAT2B//HAND2//KLF6//EGR2//CC2D1B//ELK3//EN1//ERG//ESR1//ETS1//FLI1//GRHL1//H2AFZ//IRF1//AR//JUN//MEOX2//MYB//MYBL1//NFIA//NFIB//OTX2//ZBTB7A//POU3F2//CC2D1A//RFX7//BCL11B//SMARCD2//STAT3//TGIF1//KLF10//ZNF217//BHLHE41//FOSL1//KLF7//FUBP3//HAND1//CLOCK//USP3//GADD45A//ARX//PBX3//POU4F1//TEAD1//KLF4//RBL2//REST//IRF9//E2F1//NPAS2//HMGA2//ST18//MBNL1//SLC2A4RG//FMR1//FXR1//OSR1//DZIP1//IMPDH1//REXO1//ZNF804A//TSHZ1//TOPORS//HMG20A//KLF2//ZBTB18//ZNF268//KLF12//ZNF597//RBFOX3//ZNF782//ZNF800//GLIS3//DNMT3B//DR1//E2F5//ZNF367//ZBTB9//KDM2A//MYT1L//CIC//ZFPM2//HBP1//ZDHHC1//HIVEP2//AGFG1//ZBTB41//LBR//ZBTB34//NEUROG1//SYCP3//RNF141//PIAS4//ZFR//ZFAND6//BNC2//ZNF532//BDP1//MAPK1//PHTF2//ZNF630//ZNF287//ZNF512B//TSHZ3//ZBTB26//MIER1//ARID4A//RNF4//ATXN1//SOS1//SOS2//STAT4//TMF1//TSG101//ZNF2//ZNF3//ZNF131//ZNF655//DEK//TCF7L1//LCOR//GLYR1//ZIC5//CBFB//BTAF1//ZBTB47//KMT2B//RBBP8//NUP35//NABP1//KHDRBS3//TCERG1//FASTK//CKAP4//CRKL//PABPC5//RBM33//DDX6//ZFC3H1//ELAVL2//ZCCHC24//MORC3//PAN3//FAM133B//TES//GRB2//CNOT7//NANOS1//RBM44//IPO5//ACO1//NOVA1//YTHDF2//RBM47//ZNFX1//CNOT6//RBM25//RBBP7//RPL15//SRSF2//ARHGEF28//ZMAT3//FNDC3B//CAPRIN2//BTF3//UBE2I//YWHAG//AHNAK//SNIP1//ZCCHC3//UNK//RTCA//CCT6A//QKI//SECISBP2L//G3BP2//MSN//DUS2//PAIP2//ESRP1//HIPK3//CDK6//CDK8//MAP3K2//ADCY3//CLCN5//CLCN6//PDIK1L//DGKH//DYNC1LI2//EPHA2//PIKFYVE//EPHA4//EPHA5//EPHA7//ACSL1//CDK19//DSTYK//LATS2//MKNK2//ATAD2//JAK1//KCNJ10//KDR//KIF5A//MAP3K5//MET//MYO1B//NSF//P2RY1//MINK1//CDK17//TAOK3//CMPK1//PFKP//PIK3CB//UBE2W//NAGK//PRKAA1//PRKACB//SPHK2//PAK6//FAM20C//RIMKLB//TWF1//KIF13A//MAP2K4//UBE2Z//BMPR2//STK33//HSPA13//ACTC1//TGFBR2//FYCO1//UBA5//MASTL//DGKE//RPS6KA4//ACVR1//PKDCC//RPS6KA5//STK17B//ITM2B//KIF23//ULK2//SLK//RAB12//RAB21//RAB38//ARL5A//RAB30//GLUD2//GNAQ//NKIRAS1//RHOC//RND3//SAR1B//RAB9B//DNAJC27//RASD1//RAB23//ARL15//RAB22A//RAP2C//RRAGD//RAP1B//RHEB//RASL11B//RAB28//TRERF1//MTRR//MIEF1//PGRMC2//PDE10A//RAPGEF4//PRKAR2B//HCN2//DCTPP1//UGP2//MTPN//NKX2-3//EMX2//FOXJ3//LHX6//GTF2E1//MNX1//HOXA3//HOXD1//FOXN2//LHX1//MAF//NR3C2//LHX8//NEUROD1//DMRTA1//PRDM16//SOX17//NKX2-4//SHOX//SNAPC1//ARID4B//TNF//TBL1XR1//HS3ST5//ACOX1//IDH1//DCP2//UXS1//ZEB1//TCF21//BHLHE40// |
| GO:0000979 | RNA polymerase II core promoter sequence-specific DNA binding | Molecular function | 5 | 36 | 937 | 17548 | 2.60109095221155 | 0.0410167528514512 | 0.310863811084683 | 1.38703872413626 | 0.00533617929562433 | CEBPB//GADD45A//H2AFZ//SOX11//TFAP2A// |
| GO:0097472 | cyclin-dependent protein kinase activity | Molecular function | 5 | 36 | 937 | 17548 | 2.60109095221155 | 0.0410167528514512 | 0.310863811084683 | 1.38703872413626 | 0.00533617929562433 | CDK6//CDK8//CDKN1A//CDK19//CDK17// |
| GO:0070273 | phosphatidylinositol-4-phosphate binding | Molecular function | 4 | 25 | 937 | 17548 | 2.99645677694771 | 0.0417607564151869 | 0.312514357534526 | 1.3792316435315 | 0.00426894343649947 | OSBPL5//ARFIP1//PLEKHA3//SESTD1// |
| GO:1901567 | fatty acid derivative binding | Molecular function | 4 | 25 | 937 | 17548 | 2.99645677694771 | 0.0417607564151869 | 0.312514357534526 | 1.3792316435315 | 0.00426894343649947 | ACOT7//ACBD3//ACBD5//PPARG// |
| GO:0004559 | alpha-mannosidase activity | Molecular function | 3 | 15 | 937 | 17548 | 3.74557097118463 | 0.042681359015132 | 0.312514357534526 | 1.36976176067737 | 0.0032017075773746 | MAN1A1//EDEM1//MAN2A1// |
| GO:0005243 | gap junction channel activity | Molecular function | 3 | 15 | 937 | 17548 | 3.74557097118463 | 0.042681359015132 | 0.312514357534526 | 1.36976176067737 | 0.0032017075773746 | GJC1//GJA1//GJB2// |
| GO:0030955 | potassium ion binding | Molecular function | 3 | 15 | 937 | 17548 | 3.74557097118463 | 0.042681359015132 | 0.312514357534526 | 1.36976176067737 | 0.0032017075773746 | SLC24A2//KCNA4//SLC24A3// |
| GO:0046965 | retinoid X receptor binding | Molecular function | 3 | 15 | 937 | 17548 | 3.74557097118463 | 0.042681359015132 | 0.312514357534526 | 1.36976176067737 | 0.0032017075773746 | NR4A2//PPARG//RARB// |
| GO:0003676 | nucleic acid binding | Molecular function | 249 | 4252 | 937 | 17548 | 1.09671586526925 | 0.047341896163251 | 0.344691558581872 | 1.32475435146091 | 0.265741728922092 | HMGB3//CPEB2//CPEB3//CPEB1//CPEB4//CRY2//BAHD1//NR5A2//ZNF568//MEF2D//NFE2//PPARG//RARB//SOX4//SOX11//TFAP2A//THRA//PER2//CEBPB//ZMYND11//CREBRF//E2F3//FOXF1//FOXF2//FOXC1//GATA6//PURG//JARID2//NHLH2//NME1//NR4A2//PAX9//KLF3//ZBTB4//TEF//TFAP2C//NR4A3//ARID5B//RUNX3//KAT2B//HAND2//KLF6//EGR2//CC2D1B//ELK3//EN1//ERG//ESR1//ETS1//FLI1//GRHL1//H2AFZ//IRF1//AR//JUN//MEOX2//MYB//MYBL1//NFIA//NFIB//OTX2//ZBTB7A//POU3F2//CC2D1A//RFX7//BCL11B//SMARCD2//STAT3//TGIF1//KLF10//ZNF217//BHLHE41//FOSL1//KLF7//FUBP3//HAND1//CLOCK//USP3//GADD45A//ARX//PBX3//POU4F1//TEAD1//KLF4//RBL2//REST//IRF9//E2F1//NPAS2//HMGA2//ST18//MBNL1//SLC2A4RG//FMR1//FXR1//TSHZ1//TOPORS//HMG20A//KLF2//ZBTB18//ZNF268//KLF12//ZNF597//RBFOX3//ZNF782//ZNF800//GLIS3//DNMT3B//DR1//E2F5//ZNF367//ZBTB9//KDM2A//MYT1L//CIC//ZFPM2//HBP1//ZDHHC1//HIVEP2//AGFG1//ZBTB41//IMPDH1//LBR//ZBTB34//NEUROG1//SYCP3//RNF141//PIAS4//ZFR//ZFAND6//BNC2//ZNF532//BDP1//MAPK1//PHTF2//ZNF630//ZNF287//ZNF512B//TSHZ3//ZBTB26//MIER1//ARID4A//REV3L//RNF4//ATXN1//SOS1//SOS2//STAT4//TMF1//TSG101//ZNF2//ZNF3//ZNF131//ZNF655//DEK//TCF7L1//LCOR//GLYR1//ZIC5//CBFB//BTAF1//ZBTB47//KMT2B//RBBP8//NUP35//NABP1//KHDRBS3//TCERG1//FASTK//CKAP4//CRKL//PABPC5//RBM33//DDX6//ZFC3H1//ELAVL2//ZCCHC24//MORC3//PAN3//FAM133B//TES//GRB2//CNOT7//NANOS1//RBM44//IPO5//ACO1//NOVA1//YTHDF2//RBM47//ZNFX1//CNOT6//RBM25//RBBP7//RPL15//SRSF2//ARHGEF28//ZMAT3//FNDC3B//CAPRIN2//BTF3//UBE2I//YWHAG//AHNAK//SNIP1//ZCCHC3//UNK//RTCA//CCT6A//QKI//SECISBP2L//G3BP2//MSN//DUS2//PAIP2//ESRP1//TRERF1//MTPN//NKX2-3//EMX2//FOXJ3//LHX6//GTF2E1//MNX1//HOXA3//HOXD1//FOXN2//LHX1//MAF//NR3C2//LHX8//NEUROD1//DMRTA1//PRDM16//SOX17//NKX2-4//SHOX//SNAPC1//ARID4B//TNF//TBL1XR1//DCP2//ZEB1//TCF21//BHLHE40//OSR1//DZIP1//REXO1//ZNF804A// |
| GO:0001191 | transcriptional repressor activity, RNA polymerase II transcription factor binding | Molecular function | 6 | 50 | 937 | 17548 | 2.24734258271078 | 0.04909815288901 | 0.353067604529715 | 1.30893484606847 | 0.0064034151547492 | TCERG1//RBBP8//URI1//CNOT7//BHLHE41//BHLHE40// |
| GO:0035004 | phosphatidylinositol 3-kinase activity | Molecular function | 8 | 76 | 937 | 17548 | 1.97135314272875 | 0.0493095960029926 | 0.353067604529715 | 1.30706855565969 | 0.00853788687299893 | GAB1//GRB2//PIK3CB//PIK3R3//EREG//ESR1//NRG1//MET// |
| GO:0051219 | phosphoprotein binding | Molecular function | 8 | 76 | 937 | 17548 | 1.97135314272875 | 0.0493095960029926 | 0.353067604529715 | 1.30706855565969 | 0.00853788687299893 | CRK//CRKL//GRB2//MAPK1//PIK3R3//FBXW7//PKD2//URI1// |
